# Inhibition of Bacterial Neuraminidase and Biofilm Formation by Ugonins Isolated From *Helminthostachys Zeylanica* (L.) Hook

**DOI:** 10.3389/fphar.2022.890649

**Published:** 2022-05-11

**Authors:** Abdul Bari Shah, Aizhamal Baiseitova, Jeong Ho Kim, Yong Hyun Lee, Ki Hun Park

**Affiliations:** Division of Applied Life Science (BK21 plus), IALS, Gyeongsang National University, Jinju, Korea

**Keywords:** bacterial neuraminidase, Helminthostachys zeylanica, ugonins, biofilm formation, *E. coli*

## Abstract

Bacterial neuraminidase (BNA) plays a pivotal role in the pathogenesis of several microbial diseases including biofilm formation. The aim of this study is to reveal the neuraminidase inhibitory potential of metabolites from *Helminthostachys zeylanica* (L.) Hook. which have diverse biological activities including PTP1B and *α*-glucosidase. The six ugonins (**1–6**) from the target plant showed significant neuraminidase inhibition. The inhibitory potencies were observed at a nanomolar level of 35–50 nM, which means they are 100 times more active than their corresponding mother compounds (eriodyctiol and luteolin). A detailed kinetic study revealed that all ugonins were reversible noncompetitive inhibitors. An in-depth investigation of the most potent compound **1** showed its time-dependent inhibition with the isomerization model having *k*_5_ = 0.0103 min^−1^, *k*_6_ = 0.0486 min^−1^, and *K*_i_^app^ = 0.062 μM. The binding affinities (*K*_sv_) were agreed closely with our prediction based on the inhibitory potencies. Particularly, ugonin J (**1**) blocked the biofilm formation of *E. coli* dose-dependently up to 150 µM without the inhibition of bacteria. The major compounds (**1–6**) in the extract were characterized by UPLC-ESI-Q-TOF/MS.

## Introduction

The cell surface is attached with a sugar cluster consisting of twelve sugars, which play an important role regarding cell-cell or cell-pathogen communications. Neuraminic (sialic) acid (NA) is predominant significance of cell function because of its occupation in a terminal position in the sugar cluster (Cavalcante et al., 2021). Bacterial neuraminidase (BNA) from hydrolase group of enzyme, cleaves NA with *α*-2→3 or 2→6 linkages (Nishikawa et al., 2012; Chen et al., 2018; Choi et al., 2019). Some pathogenic bacteria such as *Clostridium perfringens* recognize the neuraminic acid (NA) motif when attempting to infect a host cell; however, in the presence of NA, the infection does not proceed. NA motifs are also importantly associated with the immune system after bacterial infection. The hydrolysis of NA motifs leads to an increase in cytokines, which results in inflammation (Feng et al., 2017; Lübbers et al., 2018). Most bacteria are likely to form surface-attached biofilm communities as a survival strategy that could incur drug resistance (Chepkirui et al., 2018; Xu et al., 2020). Thus, BNA inhibitors are of considerable importance for microbial infection as well as the resistance of antibiotics.

*Helminthostachys zeylanica* (L.) Hook. is a well-known medicinal plant named “Daodi Ugon” in China (Wu et al., 2017b). Ugonin compounds are the most abundant secondary metabolites and the unique chemotaxonomy of this plant. This species belongs to the Ophioglossaceae family, which is distributed throughout Southeast Asia. Traditionally, this medicinal plant has been used to treat diabetes and inflammatory and hepatic disorders. The ugonins from this plant were found to have diverse biological activities as like antioxidative, anti-inflammatory, and enzyme inhibitions (Yamauchi et al., 2015; Chen et al., 2017; Huang et al., 2017; Chang et al., 2019; Shah et al., 2020). Their anti-inflammatory properties enable them to block the NF-κB and MAPK pathways in LPS induced mice (Wu et al., 2017a). The ugonin series are also moderate enzyme inhibitors of SARS-CoV-2 3C-like protease, PTP1B, and *α*-glucosidase (Shah et al., 2020; Chiou et al., 2021). Further investigations of ugonins led to the discovery that ugonins have significant inhibitory potential against neuraminidase and biofilm formation.

The aim of this study is to examine neuraminidase inhibition by ugonin compounds from *Helminthostachys zeylanica* (L.) Hook*.* Inhibitory mechanisms to BNA were fully characterized with detailed kinetics including slow-binding experiments and the determination of binding affinities. The representative inhibitor (**1**) was also applied to the antibiofilm experiment. The metabolites in the extract were annotated by UPLC-ESI-Q-TOF/MS.

## Materials and Methods

### Chemicals and Materials

Analytical grade chemicals used in this study were bought from Thermo Fisher Scientific (Waltham, MA, United States). Column chromatography with the packing materials octadecylsilane (ODS) silica gel (50 mm, YMC Ltd., Japan) and Sephadex LH-20 (50 mm, Amersham Pharmacia Biotech, Sweden) were used in this study. The isolation and purification of ugonins were carried out using MPLC and recycling HPLC LC-Forte/R 100 (YMC Co., Ltd., Kyoto, Japan) system equipped with a three-channel UV detector. The NMR spectra of the ugonins were acquired on a Bruker (AM 300, 500 MHz) spectrometer and a JEOL JMS-700 (JEOL Ltd., Akishima, Japan) instrument was used for MS. A SpectraMax M3 Multi-Mode Microplate Reader (Molecular device, United States) was used for the enzyme inhibition assays and biofilm formation assessment.

### Plant Material and Isolation

The rhizome of *Helminthostachys zeylanica* (L.) Hook*.* was purchased at a local market in Taiwan in 2018 (lane 224, Xichang St, Wanhua District, Taipei) and has been identified by Assoc. Prof. Dr. Mohd Azlan Nafiah, faculty of science and mathematics, Universiti Pendidikan Sultan Idris 35900 Tg. Malim, Perak, Malaysia, with voucher number TM1054. In the first step, the dried and powdered rhizome of *Helminthostachys zeylanica* (L.) Hook*.* (1 kg) was soaked in 10 L of MeOH to afford 7% extraction yield (70 g). The methanol extract was dissolved in water and then fractionated in order of solvent polarities with hexane, chloroform, ethyl acetate and n-butanol. The phenolic compounds enriched ethyl acetate fraction (13 g) was subjected to silica gel (100 × 300 mm, 230–400 mesh, 720 g) column chromatography. It was eluted gradiently with *n*-hexane and ethyl acetate (20:1 to 1:2) to afford 10 subfractions (A-J). Based on the TLC results, subfractions (B-E) were rechromatographed on Sephadex LH-20 and eluted with methanol to give four different subfractions (B1-7, C1-10, D1-15, E1-10). Subfractions B and C were passed in different solvents systems (water and methanol) to recycling HPLC of the ODS gel that gives us compounds **1** (50 mg), **2** (13 mg), and **3** (10 mg). Subfractions D and F were subjected to MPLC (250 mm × 30 mm, S-10 l m, 12 nm, YMC) and eluted by gradually increasing the MeOH yielding compounds **4** (14 mg), **5** (12 mg), and **6** (13 mg).

### Bacterial Neuraminidase Inhibition Assay

The bacterial neuraminidase (BNA) assay was conducted on the SpectraMax M3 Multi-Mode Microplate Reader, LLC. (Molecular Devices, San Jose, CA, United States) according to a previous method (Choi et al., 2019). The fluorescence was measured with an emission wavelength of 450 nm and an excitation wavelength of 365 nm. First, a 96-well black immuno-microplate (SPL Life Science, Korea) was taken and each individual well was filled with 20 µl of 1 mM substrate (4-methylumbelliferyl-N-acetyl-α-_D_-neuraminic acid sodium salt hydrate) aqueous solution mixed with 160 µl of 50 mM sodium acetate buffer (pH 5.0), after which 10 µl of the inhibitors with 10 µl BNA (0.2 units/ml) were added. The concentration of inhibitor that leads to 50% activity loss (IC_50_) was calculated by the following equation:

$$\text{Activity }\left(\text{\%}\right)\;=\;[1\;+\;(\left[\text{I}\right]\text{ / I}{\text{C}}_{50})]\text{× }100$$ (1)

### Bacterial Neuraminidase Kinetic Assay

The enzyme kinetic assay was performed in the presence of different concentrations of the substrate (0.5, 1, and 2 mM) and inhibitors according to previous research work (Choi et al., 2019). Sigma Plot® software (Systat Software, Inc., Chicago, IL, United States) was used to measure the variables from the curves. The different constants and values such as the Michaelis-Menten (*K*_m_) constant and maximum velocity (*V*_max_) were obtained from Lineweaver-Burk plots and the dissociation constants of the enzyme and inhibitors (*K*_i_) were obtained from Dixon plots.

### Time-Dependent Assays and Progress Curves

The assays were carried out by using BNA (0.2 unit/ml), and 1 mM substrate in 50 mM sodium acetate buffer (pH 5.0). The enzyme activity was measured for 30 min using the SpectraMax M3 Multi-Mode Microplate Reader. The kinetic parameters and time-dependent inhibition of BNA were determined by recording progress curves at various inhibitor concentrations using the original substrate concentration. Nonlinear regression SigmaPlot® (SPCC Inc., Chicago, IL) was used to analyze the data that provide the different parameters of each curve: *v*_i_ (initial velocity), *v*_s_ (steady-state velocity), *k*_obs_ (apparent first-order rate constant for the transition from *v*_i_ to *v*_s_), A (absorbance at 405 nm), and *K*_i_ app according to equations (2) and (3) (Li et al., 2018; Kim et al., 2020).

$$A=v_{s}t+\left(v_{i}-v_{s}\right)[1-\text{exp}\left(-k_{obs}t\right)/k_{obs}$$ (2)

$$K_{obs}=k_{6}+\left[\frac{k_{5}\times \left[\text{I}\right]}{K_{i}^{app}+\left[\text{I}\right]}\right]$$ (3)

### Measurement of the Binding Affinity of the Enzyme

The fluorescence quenching experiment was performed according to previous work (Wang et al., 2019). First, to 96-well black immune plates were taken and then subsequently 180 µl of buffer and 10 µl of 0.5 unit/ml neuraminidase were added. Then, different concentrations (0, 0.15625, 0.3125, 0.625,1.25, 2.5 µM) of inhibitors were added. The fluorescence emission spectra were recorded in the range of 300–400 nm, with the emission slits of the spectrometer corrected at 2 nm at an excitation wavelength of 260 nm.

### Bacterial Strains and Culture Conditions

*Escherichia coli* was used in the study. All experiments were conducted in Difco™ Nutrient Broth (DNB) medium at 37°C. The *E. coli* was removed from glycerol stock at −80 C and transferred to the DNB plate. Next, in a 250 ml flask, a fresh single colony was inoculated into DNB (25 ml) medium and cultured at 37 C with 250 rpm for 24 h. Using the SpectraMaxM3 Multi-Mode Microplate Reader cell growth was found in the presence of different concentrations of compounds at optical densities of 600 nm. All experiments were conducted with at least three independent cultures (Cho et al., 2013).

### Antibiofilm Assays

A 96-well microtiter plate (polystyrene) was used to measure the anti-biofilm activity as described previously (Mohanta et al., 2020). First, 180 μl of DNB and 10 μl of the test pathogens (OD = 1.0, 600 nm) were added to individual wells. Then, 10 μl solutions of ugonin J with different concentrations was added to each well and the contents of each well were mixed thoroughly. The prepared plate was then incubated for 24 h in a static condition at 37°C. After incubation, the supernatant in the wells in the plate was discarded and the residue washed with phosphate buffered saline (pH 7.2) to remove the free-floating non-adherent cells from the wells in the plate. The plate was then air-dried for about 45 min before the bacteria in the wells were fixed with 2% (w/v) sodium acetate. This was followed by flooding the wells with crystal violet stain (0.1%, w/v) and incubation in the dark for 30 min. After this, the wells in the plate were washed with sterile deionized water to remove all dye and allowed to dry. After drying, each well in the plate was filled with 200 μl of ethanol (95%, v/v) and the absorbance of this solution was measured at 620 nm using the SpectraMax M3 Multi-Mode Microplate Reader. All assays were conducted in triplicate and the mean ± standard deviation was calculated. The percent inhibition of biofilm formation was obtained through the following equation.

$$\%\;biofilm\;inhibition=\left[1-\left(\frac{OD620\;of\;cell\;treated\;with\;UgoninJ}{OD620\;of\;Control}\right)\times \;100\right]$$ (4)

### UPLC-ESI-Q-TOF/MS Analysis

The methanolic extract of *Helminthostachys zeylanica* (L.) Hook. root was investigated for the metabolites using UPLC-ESI-Q-TOF/MS. The different peaks of compounds on the base peak intensity (BPI) chromatogram were determined by an Acquity UPLC BEH C_18_ column (2.1 mm × 100 mm, 1.7 μm; Waters Corp., MA, United States). A gradient elution system consisting of water with 0.1 % formic acid (solvent A) and acetonitrile (solvent B) were used as the mobile phase. The gradual elution conditions were: 0.00–1.00 min, 99.0% A; 1.00–10.00 min, 0% A, 10.00–15.00 min, 99.0% A, at a flow rate of 0.35 ml/min. The voltage of the capillary and sample cone was set to 3 kV and 30 V, respectively. The gas flow through the cone was 800 L/h and desolvation was carried out at 30 L/h, and the temperature of desolvation and the ion source were 400 and 100°C, respectively. Positive mode [(M + H)^+^] at *m/z* 50–1,500 was used for mass spectra using leucine-enkephalin as the reference as a lock mass (556.2771 Da). Unifi software (Waters) was used for the identification and analysis of individual compounds.

## Results and Discussion

### Structural Elucidation

**FIGURE 1:**
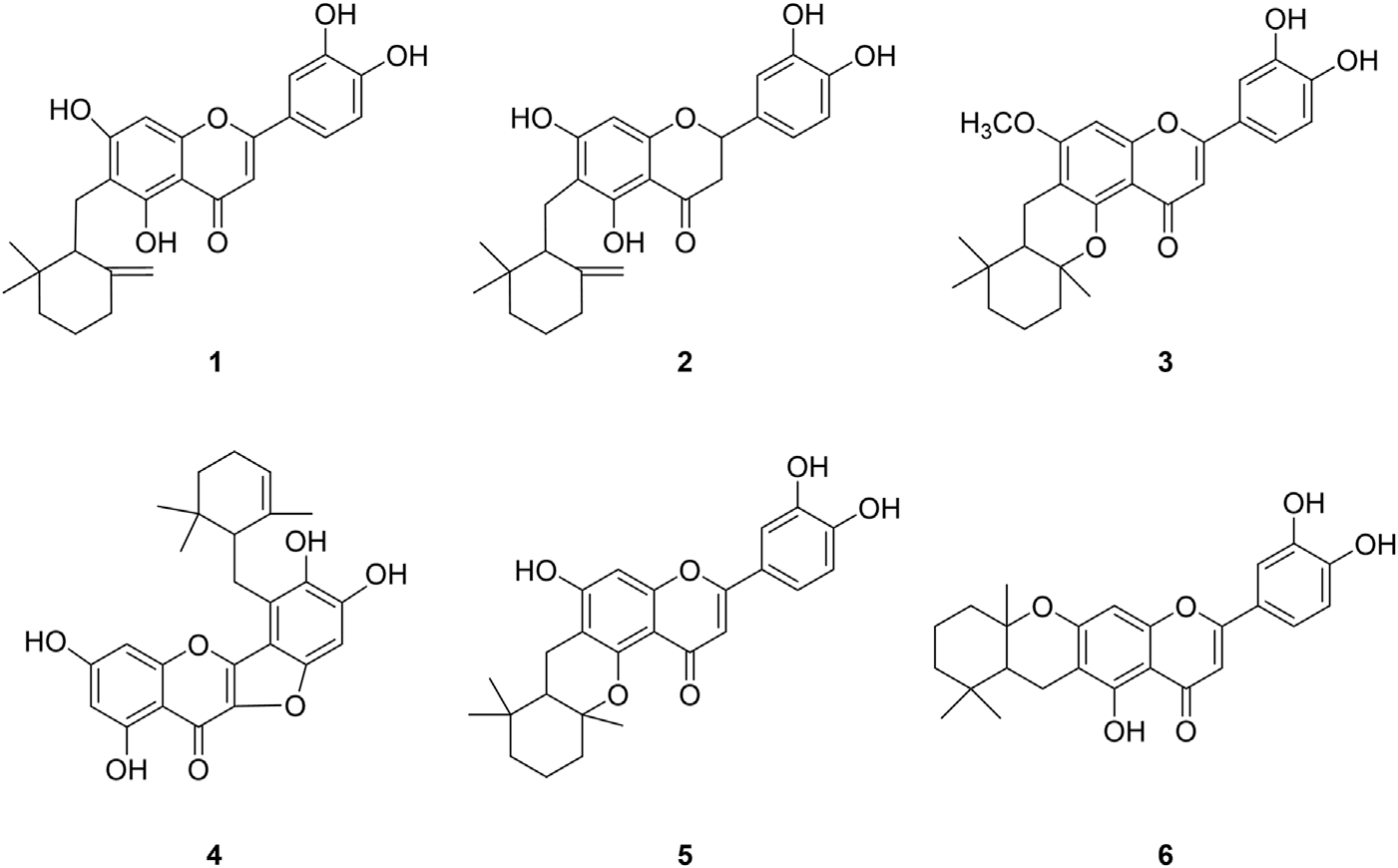
Chemical structures of isolated bacterial neuraminidase inhibitors from *Helminthostachys zeylanica (L.) Hook.*

The study was starting with the methanol extract of *Helminthostachys zeylanica* (L.) Hook. roots that exhibited significant BNA inhibitory activity with an IC_50_ of 35 μg/ml. We purified the compounds that were found to have BNA inhibitory activity (Figure 1). Repeated column chromatography of the extract yielded six ugonins (**1–6**) in accordance with previous the literature. The isolated compounds were identified as ugonin J (**1**), 2-(3,4-dihydroxyphenyl)-6-[(2,2-dimethyl-6 methylenecyclohexyl) methyl]-5,7-dihydroxy-chroman-4-one (**2**), ugonin L (**3**), ugonin M (**4**), ugonin S (**5**), and ugonin U (**6**) by comparing their spectroscopic data (Supplementary Figures S2–S4) to those previously reported (Huang et al., 2009).

### Bacterial Neuraminidase Inhibition

**FIGURE 2:**
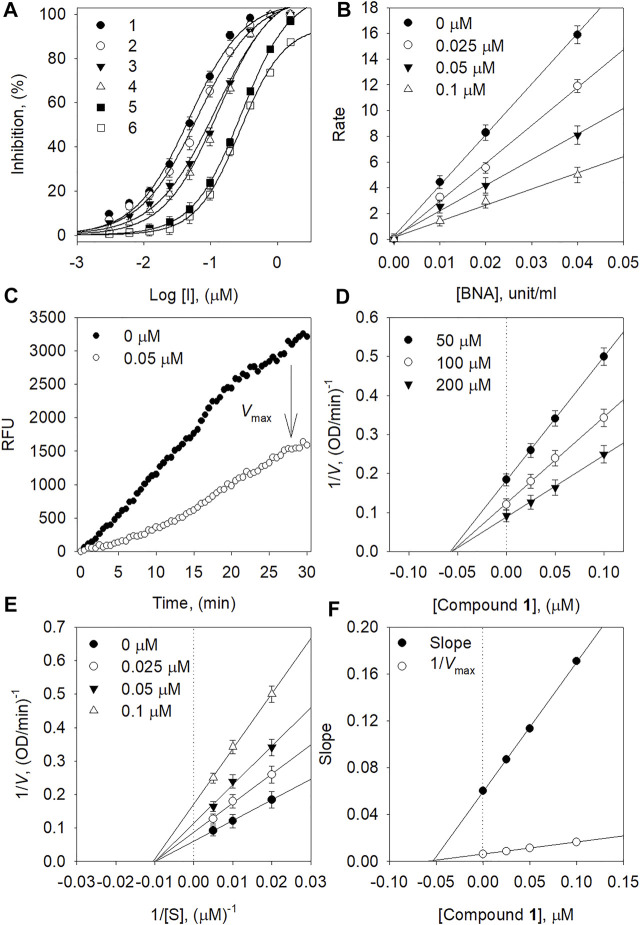
**(A)** Dose-dependent inhibition effect of compounds on BNA **(B)** The catalytic activity of BNA as a function of enzyme concentration at different concentrations of compound **1**. **(C)** Inhibition effect of the substrate by BNA with and without **1. (D)** Dixon plots and **(E)** Lineweaver-Burk of BNA inhibition of compound **1**. **(F)** Secondary plots of the Lineweaver–Burk plot, 1/*V*_max_ or slope *vs*. various concentrations of inhibitor.

The BNA inhibitory capacities of the isolated ugonins (**1–6**) were evaluated by the release of 4-methylumbelliferone from 2′-(4-methylumbelliferyl)-α-_D_-*N*-acetylneuraminic acid (Choi et al., 2019). Quercetin, a known inhibitor from polyphenols was used as a positive control (IC_50_ = 20.4 µM). All of the ugonins (**1–6**) had a dose-dependent inhibitory effect on BNA, with maximal activities observed at 10 µM (Figure 2A). As the concentration of the inhibitors increased, the residual enzyme activity rapidly diminished. Compounds **1-6** had significant inhibitory capacities toward BNA activity, with IC_50_ values of 0.05∼0.35 µM (Table 1). All compounds inhibited BNA activity at the nanomolar level, which might be considered as the most marked inhibitor in the polyphenol series.

**TABLE 1:** Inhibition of bacterial neuraminidase (BNA) by compounds isolated from *H. zeylanica*.

Compounds	IC_50_ ^a^ (μg/ml, μM)	Kinetic mode (*K*_i_ ^b^, μM)
**1**	0.05	Noncompetitive (0.05)
**2**	0.07	Noncompetitive (0.06)
**3**	0.14	Noncompetitive (0.13)
**4**	0.15	Noncompetitive (0.12)
**5**	0.28	Noncompetitive (0.25)
**6**	0.35	Noncompetitive (0.29)
**Eriodictyol**	17.8	NT^c^
**Luteolin**	4.4	NT
**Quercetin** ^d^	20.4	NT

a All compounds were examined in a set of experiments repeated three times and IC_50_ values of compounds represent the concentrations in μM (μg/ml for extracts) that caused 50% loss of enzyme activity.

b Values of inhibition constant.

c NT is not tested.

d Quercetin is positive control.

To clarify the structure-activity relationship (SAR), the isolated ugonins (**1–6**) were compared to their parent compounds (luteolin and eriodyctiol). Alkyl substitution on the C6 position within the parent compounds seems to increase the BNA inhibitory capacity greatly. The marked potency of **1** relative to luteolin suggests that the cyclohexylmethyl motif function is paramount to inhibitor activity; **1** (IC_50_ = 0.05 µM) *vs*. luteolin (IC_50_ = 4.4 µM). Similar SAR results were also found between compound **2** and eriodyctiol. The inhibitive ability of **2** (IC_50_ = 0.07 µM) bearing the cyclohexylmethyl motif was 250-fold greater than that of eriodyctiol (IC_50_ = 17.8 µM). The compounds (**3, 5,** and **6**) were derived from the cyclization of the cyclohexylmethyl motif with C5-OH or C7-OH. They also showed much improved inhibitory capacities compared to parent compounds with IC_50_ values of 0.28, 0.14, and 0.35 µM, respectively.

The mechanism of inhibitory action by the isolated ugonins was subsequently studied. All ugonins displayed a similar relationship between BNA activity and concentration. Figure 2B shows a plot of the initial velocity according to the BNA concentration in the presence of different concentrations of **1**. Increasing the inhibitor concentration resulted in lines with a lower gradient. Thus, compound **1** was found to be a reversible inhibitor because of the family of straight lines passing through the origin. The inhibition of BNA by compound **1** is illustrated in Figure 2C representatively.

In the kinetic analysis conducted by using Lineweaver-Burk plots in Figure 2E, the x-intercept (−1/*K*_m_) was unaffected by the concentration of compound **1**, whereas the 1/*V*_max_ values became more positive. This indicated that *V*_max_ decreased without changing *K*_m_ (Figure 2F). Thus, compound **1** was a noncompetitive inhibitor to the BNA enzyme. The *K*_i_ values in Table 1 were calculated from Dixon plots (Figure 2D) that were obtained by plotting 1/*V* vs. [I] with varying concentrations of the substrate. The most potent inhibitor **1** has 50 nM of *K*_i_. All Lineweaver-Burk and Dixon plots of the other ugonins are displayed as Supplementary Information (Supplementary Figures S21, S22).

**FIGURE 3:**
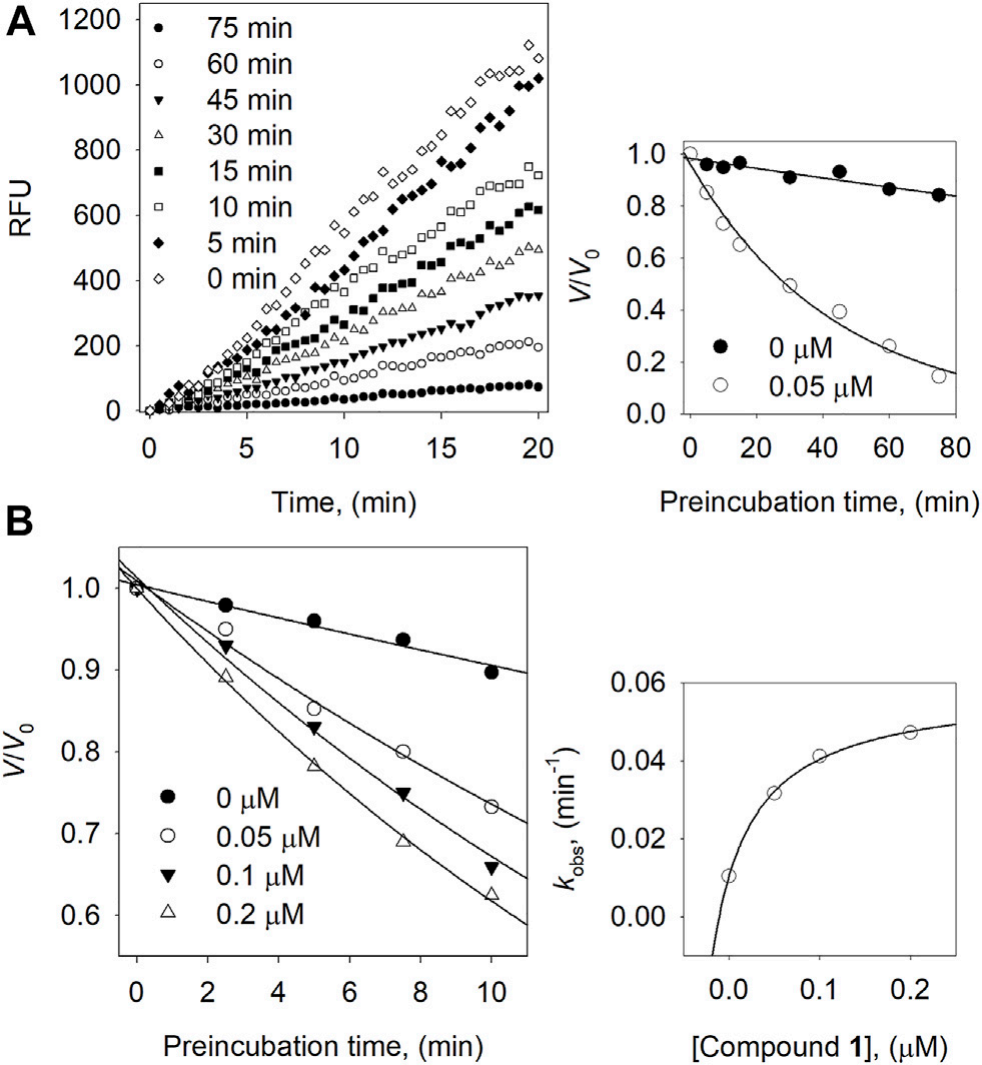
**(A)** Slow-binding inhibition at different preincubation time (◇: 0; ◆: 5; □: 10; ■: 15; △: 30; ▼: 45; ○: 60; ●: 75 min) for compound **1** at 0.05 μM. Inset: Inhibition as a function of preincubation time for compound **1**. **(B)** Time course of the inactivation of BNA compound **1**. Inset: plot of *k*_obs_ on the dependence on different concentrations of compound **1**.

We then carried out a full kinetic characterization of inhibitor **1**. Figure 3 depicts the slow-binding inhibition for the hydrolysis of neuraminic acid catalyzed by the BNA enzyme in the presence of **1**. Figure 3A shows initial velocity for substrate hydrolysis as a function of the preincubation time of 0∼75 min at low concentration (0.05 µM) of compound **1**. The initial velocities for substrate hydrolysis were lowered significantly by the preincubation time and were consistent on a typical progress curve for slow-binding inhibition. Increasing the concentration of compound **1** led to a decrease in both the initial velocity (*v*_i_) and steady-state rate (*v*_s_).

Furthermore, using different concentrations of **1** result in the progress curve that yields the values of *v*_i_, *v*_s_, and *K*_obs_ by fitting to eqn. 2. The different parameters were attained from the plots with the help of eqn. 3 which gives *k*_5_ = 0.0103 min^−1^, *k*_6_ = 0.0486 min^−1^, and *K*_i_^app^ = 0.062 μM. Likewise, the dependence of *K*_obs_ on the inhibitor concentration displays a hyperbolic pattern after fitting the results to eqn. 3. Nonetheless, we obtained a hyperbolic pattern representing that **1** inhibited the BNA by the quick formation of an enzyme complex (E·I), which is further converted to a modified enzyme complex (E^*^·I) with a slow isomerization process, as shown in Figure 3B.

### Binding Affinity of Compounds to BNA Enzyme

**FIGURE 4:**
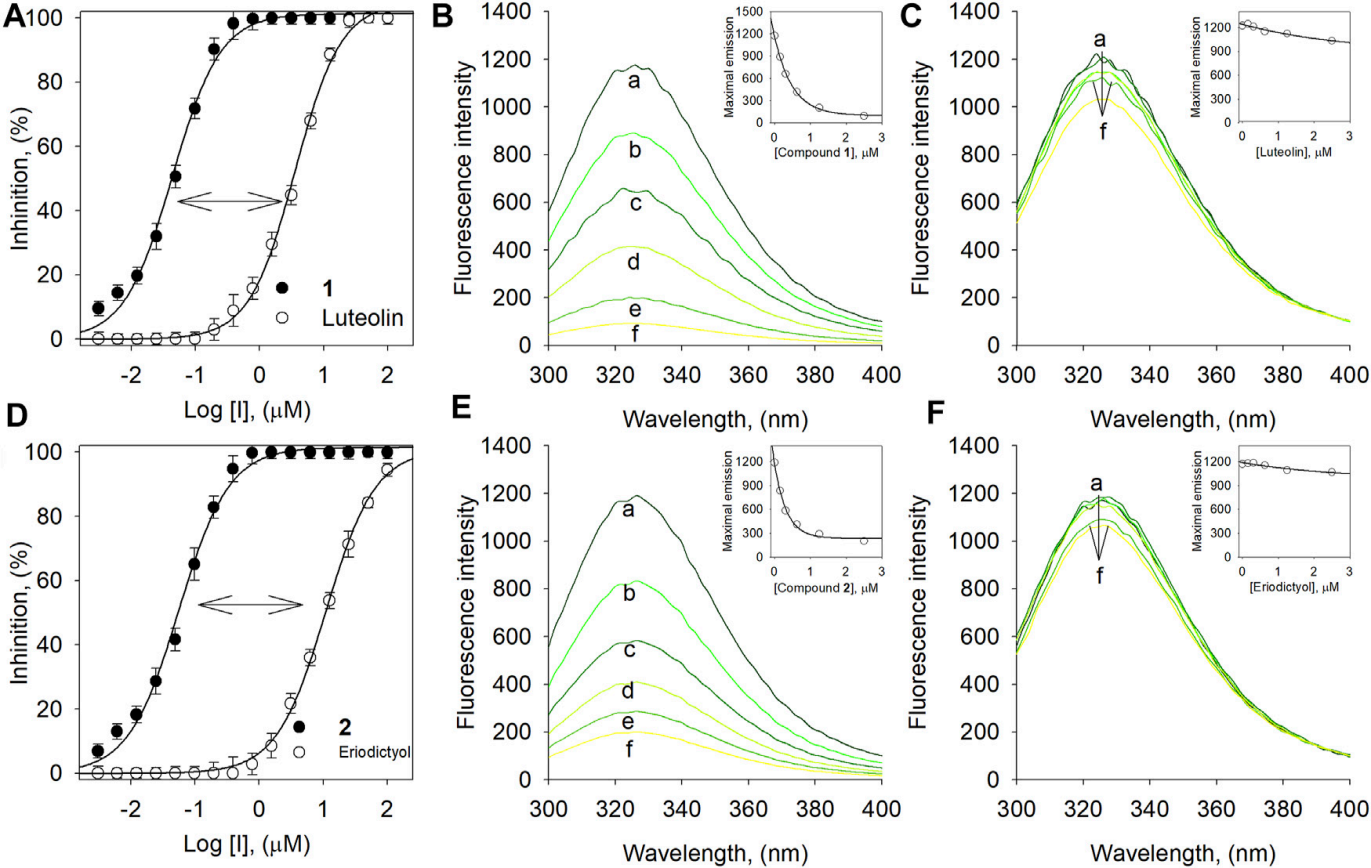
**(A)** Dose-dependent inhibition effect of compound **1** *vs*. luteolin on BNA. **(B)** The fluorescence emission spectra of BNA at different concentrations of **1** and **(C)** luteolin. **(D)** Dose-dependent inhibition effect of compound **2** *vs*. eriodictyol on BNA. **(E)** The fluorescence emission spectra of BNA at different concentrations of **2** and **(F)** eriodictyol (0, 0.16, 0.31, 0.63, 1.25, and 2.5 μM for curves a→f).

We evaluated the binding affinities to confirm that inhibitory potencies originated between by interaction between inhibitors and the BNA enzyme (Figures 4A–D). The binding affinities were measured by the fluorescence (FS) quenching effect of intrinsic protein. The BNA enzyme has intrinsic FS properties originating from the nine tryptophan, twenty-two tyrosine, and eight phenylalanine residues (Supplementary Figure S24) (Wang et al., 2019). This intrinsic FS would be changed when BNA interacts with inhibitors (ligand). The FS intensity was measured at emission wavelengths ranging from 300 to 400 nm, where no significant emission was observed from the assay solution.

Compound **1** bearing the luteolin skeleton showed a dose-dependent FS reduction in the range 1–3 µM. As shown in Figure 4B, at a concentration of 3 µM **1** underwent complete FS reduction. In contrast, the mother compound, luteolin, did not undergo FS reduction up to concentrations of 3 µM (Figure 4C). This phenomenon was in agreement with the inhibitory potencies (Figure 4A); **1** (IC_50_ = 0.05 µM) *vs.* luteolin (IC_50_ = 4.4 µM). This distinctive difference toward FS reduction was also observed between compound **2** (IC_50_ = 0.07 µM) and its corresponding mother skeleton, eriodictyol (IC_50_ = 17.8 µM) as shown in Figures 4D–F. This might suggest that the cyclohexyl methyl motif in **1** and **2** would be a critical functionality for binding to the BNA enzyme and for the inhibitory potencies. The *K*_SV_ values were calculated by the following Stern-Volmer equations (Baiseitova et al., 2021).

$$F_{\text{0}}\;-\;F\;=\text{ 1 }+\;K_{\text{SV}}\left[\text{Q}\right]$$ (5)

$$\text{log}\left[(F_{\text{0}}\;-\;F)/F\right]\;=\text{ log}K_{\text{A}}\;+\;n\text{log}[Q{]}_{\text{f}}$$ (6)

Where *F*_0_ is the fluorescence intensity in the absence of the quencher and *F* represents the presence of the quencher. The free ugonin concentration is represented by *Q*_f_, *n* is the number of binding sites, and *K*_A_ is the binding constant for the assessible fluorophores. The Stern-Volmer quenching constant (*K*_SV_) of compounds **1-6** increased in proportion to the order of their inhibitory potencies from **1** (4.77× 10^6^ L mol^−1^) to **6** (0.05× 10^6^ L mol^−1^) as shown in Tables 1, 2 (Supplementary Figure S23). The results also indicated that the BNA enzyme mostly uses a single binding site for the ugonin derivatives.

**TABLE 2:** Fluorescence quenching effects of compounds **1–6** on BNA.

Compounds	*K*_SV_ ^a^ (× 10^6^ L mol^−1^)	*K*_A_ ^b^ (× 10^6^ L mol^−1^)	*N* ^c^
1	4.77	3.54	1.30
2	2.53	1.34	1.01
3	0.97	1.02	1.01
4	0.81	0.85	1.13
5	0.07	0.06	1.24
6	0.05	0.03	1.44

a *K*_SV_, is the Stern-Volmer quenching constant.

b *K*_A_, is the binding constant for the assessible fluorophores.

c *n* is the number of binding sites.

### Cell Growth and Biofilm Inhibition

**FIGURE 5:**
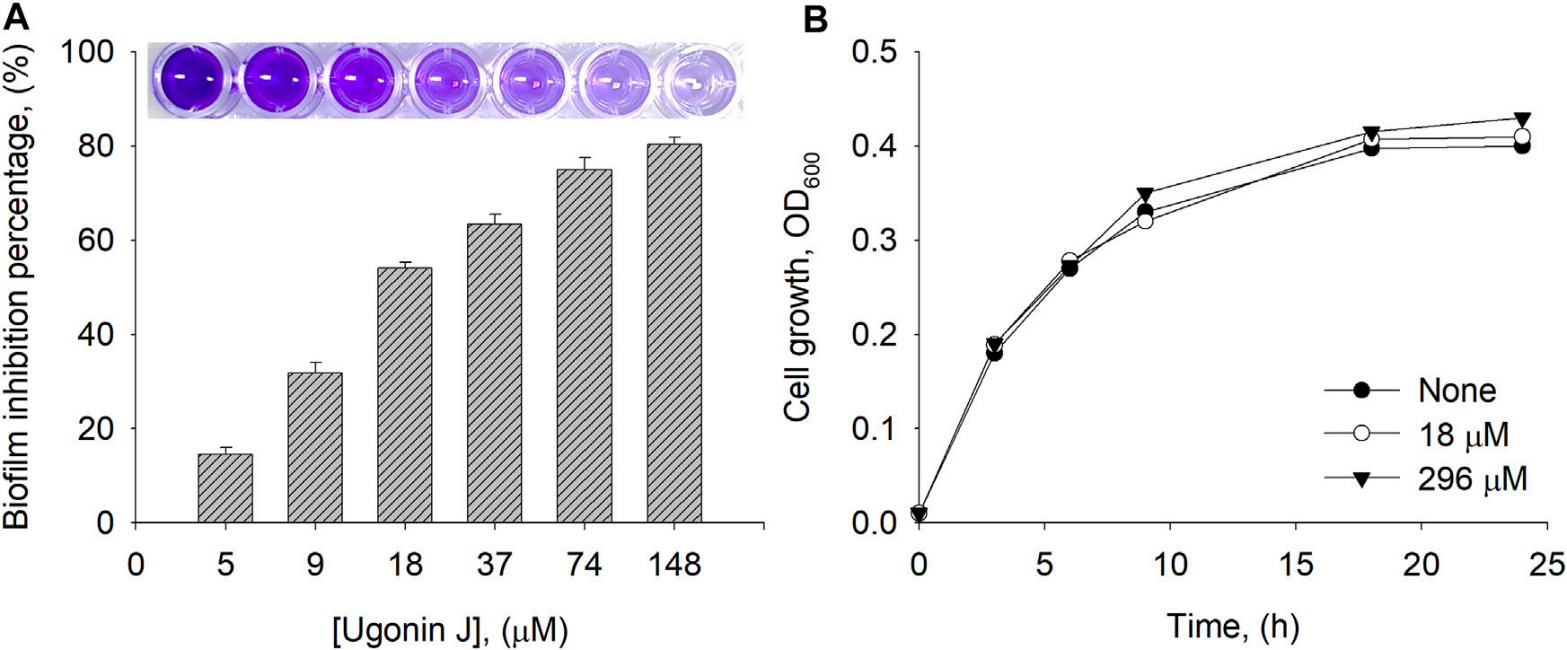
**(A)** Biofilm inhibition percentage by compound **1** at different concentrations **(B)** Cell growth of *E. coli* measured at 0, 18 and 296 µM of **1**.

Bacterial biofilms are a serious health concern owing to their abilities to tolerate antibiotics, host defense systems, and other external stresses (Sharma et al., 2019). It is generally accepted that neuraminidase participates in biofilm production together with quorum sensing. Our most potent and abundant compound **1** was tested for its ability to prevent biofilm formation. The crystal violet staining method was used for the detection of biofilm production. To investigate biofilm inhibition, *E. coli* was cultured overnight with or without inhibitor **1** in a 96-well plate. Inhibitor **1** showed no growth inhibition up to a concentration of 296 µM compared to the control (Figure 5B). As shown in Figure 5A, inhibitor **1** inhibited the production of *E. coli* biofilm dose-dependently with 50% inhibition at 18 µM.

### UPLC-ESI-Q-TOF/MS Analysis

**FIGURE 6:**
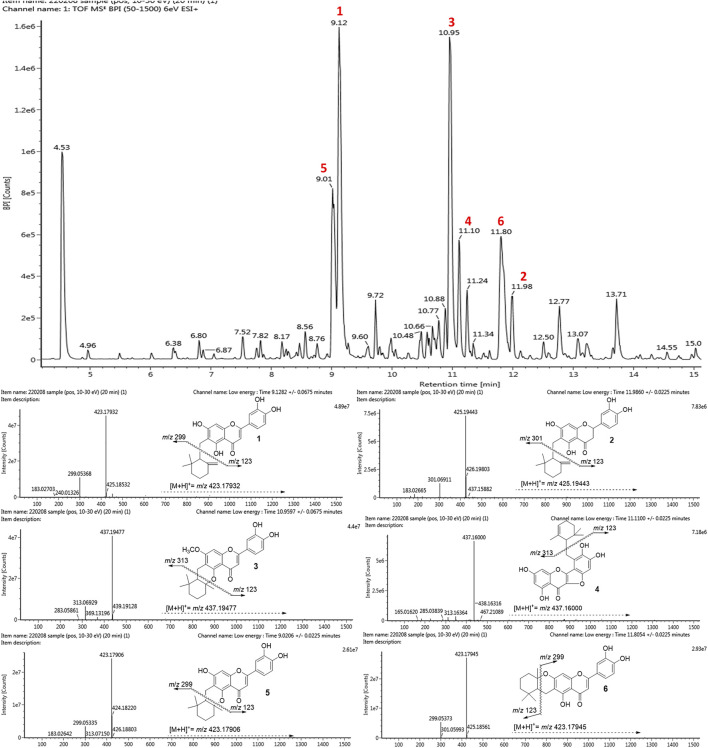
The UPLC-ESI-Q-TOF/MS base peak intensity (BPI) chromatogram of *Helminthostachys zeylanica (L.) Hook.* methanolic extract and mass spectra of six representative flavonols: ugonin J (**1**), 2-(3,4-dihydroxyphenyl)-6-[(2,2-dimethyl-6-methylenecyclo-hexyl) methyl]-5,7-dihydroxy-chroman-4-one (**2**), ugonin L (**3**), ugonin M (**4**), ugonin S (**5**), and ugonin U (**6**).

A comparative analysis is important to establish the abundance of bioactive compounds in a practical context. The methanolic extract of native roots was analyzed by using UPLC-ESI-Q-TOF/MS. The BPI chromatogram with positive ESI mode showed the complete separation of phenolic metabolites including major and minor peaks within 15 min (Figure 6). Supplementary Table S1 (Supplementary Information) provides the retention times, molecular ions [(M + H)^+^], and elementary compositions of the identified phenolic compounds. All peaks represented molecular ions with masses consistent with those of the isolated compounds [M + H]^+^ at *m/z* 423.17932 (**1**, peak 2, *t*_R_ = 9.12 min), *m/z* 425.19443 (**2**, peak 6, 11.98 min), *m/z* 437.19477 (**3**, peak 3, 10.95 min), *m/z* 437.16000 (**4**, peak 4, 11.10 min), *m/z* 423.17906 (**5**, peak 1, 9.01 min) and *m/z* 423.17945 (**6**, peak 5, 11.80 min). The fragmentation analysis of the mass data revealed that all compounds had molecular ions as well as corresponding daughter ions that are equivalent to the mother flavonoid skeleton after cleavage of each cyclohexylmethyl motif. In particular, the isolated BNA inhibitors (**1–6**) were proven to be the most abundant metabolite in the *Helminthostachys zeylanica* (L.) Hook. root part.

## Conclusion

This study found ugonin derivatives (**1–6**) as a lead structure for BNA inhibition from the root part of *Helminthostachys zeylanica* (L.) Hook., a Chinese traditional plant. All six compounds showed extremely high inhibition with the nanomolar level of IC_50_s, which are 100–300 fold more potent than their corresponding mother compounds. The inhibitory mechanism was fully characterized to be a reversible noncompetitive inhibitor with time-dependent behaviour. This time dependence of (**1**) is associated with the isomerization model with the parameters: *k*_5_ = 0.0103 min^−1^, *k*_6_ = 0.0486 min^−1^, and *K*_i_^app^ = 0.062 μM. The binding affinities (*K*_SV_) corresponded with the order of inhibitory potencies (IC_50_). Particularly, compound **1** showed a dose-dependence (0∼148 μM) and significant inhibitory activity to biofilm formation. We finally disclosed that all the BNA inhibitors (**1–6**) are the most abundant metabolites in the root part by UPLC-ESI-Q-TOF/MS. The target ugonins (**1–6**) could be expected a synergy effect together with previously reported PTP1B and *α*-glucosidase inhibitions.

## Data Availability

The original contributions presented in the study are included in the article/Supplementary Materials, further inquiries can be directed to the corresponding author.
