# Supplementary material for: Inhibition of Bacterial Neuraminidase and Biofilm Formation by Ugonins Isolated From Helminthostachys Zeylanica (L.) Hook
Source: Front Pharmacol. 2022 May 11;13:890649. doi: 10.3389/fphar.2022.890649 (PMC9130766; doi:10.3389/fphar.2022.890649)
Supplement: Supplementary file 1 [file DataSheet1.DOCX]

**SUPPLEMENTARY MATERIAL**

**Inhibition of bacterial neuraminidase and biofilm formation by ugonins isolated from *Helminthostachys zeylanica***

Abdul Bari Shah ^a^, Aizhamal Baiseitova ^a^, Jeong Ho Kim ^a^, Yoon Hyun Lee ^a^, and Ki Hun Park ^a,^ *

*^a^ Division of Applied Life Science (BK21 plus), IALS, Gyeongsang National University, Jinju 52828, Republic of Korea*

**Correspondence to:**

Prof. Dr. Ki Hun Park, Division of Applied Life Science (BK21 plus), IALS, Gyeongsang National University, Jinju 52828, Republic of Korea. Email: khpark@gnu.ac.kr Phone: +82-55-772-1965; fax: +82-55-772-1969 (K. H. Park)

**List of Contents:**

^13^C NMR spectroscopic data of compounds **1-6** S2

1D, 2D NMR, MS and HRMS data of compounds **1-6** S4

Lineweaver Burk and Dixon plots of compound **2-6** S15,16

The fluorescence spectra of compounds **3**, **4**, **5** & **6**, and the correlation between inhibitory potencies (IC_50_s) and Stern–Volmer constant (*K*_SV_) S17

Fluorescence residues of neuraminidase from C. perfrigens S18

UPLC-ESI-Q-TOF/MS characterization of compounds isolated from *H. zeylanica* S19

**^13^C NMR spectroscopic data of compounds:**

***Ugonin J*** (**1**)^13^C NMR (125 MHz, MeOD) *δ*  (C-9), 24.1 (C-15), 28.6 (C-13), 29.4 (C-12), 31.2 (C-16), 35.2 (C-14), 35.6 (C-11), 52.9 (C-10), 93.8 (C-8), 104.1 (C-3)  (C-4a),  (C-18),  (C-6), 113.9 (C-2ʹ), 116.5 (C-5ʹ), 119.9 (C-6ʹ), 123.8 (C-1ʹ), 146.3 (C-3ʹ), 149.8 (C-4ʹ), 150.5 (C-17), 156.3 (C-8a), 160.2 (C-7), 162.8 (C-5), 164.7 (C-2), 182.9 (C-4)

***2-(3,4-dihydroxyphenyl)-6-((2,2-dimethyl-6-methylenecyclo-hexyl)methyl)-5,7-dihydroxy-chroman-4-one*** (**2**)^13^C NMR (125 MHz, acetone-*d*_6_) *δ*  (C-9),  (C-13), 27.8 (C-16), 28.6 (C-17), 31.8 (C-12), 35.2 (C-14), 35.8 (C-15), 43.8 (C-3), 53.1 (C-10), 80.0 (C-2), 95.1 (C-8), 102.9 (C-4a), 109.5 (C-6), 109.5 (C-18),  (C-2ʹ),  (C-6ʹ), 119.2 (C-5ʹ),130.9 (C-1’) 146.0 (C-4ʹ), 146.4 (C-3ʹ), 150.7 (C-11), 161.8 (C-5), 162.9 (C-8a), 165.3 (C-7), 197.3 (C-4)

***Ugonin*** ***L*** (**3**)^13^C NMR (125 MHz, MeOD)* *  (C-9), 19.9 (C-16), 20.7 (C-18), 20.9 (C-13), 32.4 (C-11), 34.4 (C-15), 40.3 (C-14), 42.5 (C-12), 47.8 (C-10), 56.6 (7-OCH_3_), 80.0 (C-17), 91.7 (C-8), 106.8 (C-3)  (C-4a),  (C-6), 113.9 (C-2ʹ), 116.4 (C-5ʹ), 119.8 (C-6ʹ), 123.7 (C-1ʹ), 146.9 (C-3ʹ), 150.4 (C-4ʹ), 154.8 (C-5), 159.5 (C-8a), 163.4 (C-7), 163.5 (C-2), 180.3 (C-4)

***Ugonin M*** (**4**)^13^C NMR (125 MHz, MeOD) *δ*  (C-13), 24.9 (C-18), 27.2 (C-16), 29.1 (C-17), 30.5 (C-9), 30.7 (C-14), 33.9 (C-15), 49.4 (C-10), 95.5 (C-8), 96.6 (C-5ʹ), 100.2 (C-6), 105.4 (C-4a), 110.6 (C-1ʹ)  (C-12),  (C-2ʹ), 135.0 (C-2), 137.7 (C-11), 143.7 (C-3ʹ), 151.4 (C-6ʹ), 152.4 (C-4ʹ), 153.0 (C-3), 158.8 (C-8a), 164.0 (C-5), 165.0 (C-7), 170.8 (C-4)

***Ugonin S*** (**5**)^13^C NMR (125 MHz, MeOD) *δ*  (C-13), 19.3 (C-9), 21.7 (C-16), 27.1 (C-18), 32.7 (C-17), 35.0 (C-15), 39.9 (C-12), 42.6 (C-14), 44.1 (C-10), 78.1 (C-11), 94.8 (C-8), 106.7 (C-6), 108.4 (C-3), 108.5 (C-4a),  (C-2ʹ),  (C-5ʹ), 119.7 (C-6ʹ), 124.0 (C-1ʹ), 146.9 (C-4ʹ), 147.9 (C-3ʹ), 157.2 (C-5), 159.2 (C-8a), 161.9 (C-7), 163.3 (C-2), 180.5 (C-4)

***Ugonin U*** (**6**)^13^C NMR (125 MHz, MeOD) *δ*  (C-9), 17.62 (C-13), 20.31 (C-16), 25.92 (C-18), 31.24 (C-17), 33.49 (C-15), 38.78 (C-12), 40.97 (C-14), 42.90 (C-10), 77.23 (C-11)  (C-8),  (C-3),  (C-4a), 106.05 (C-6), 112.77 (C-2ʹ), 115.36 (C-5ʹ), 118.94 (C-6ʹ), 122.38 (C-1ʹ), 145.64 (C-3ʹ), 149.60 (C-4ʹ), 155.52 (C-8a), 158.06 (C-5), 161.28 (C-7), 164.99 (C-2), 182.63 (C-4)


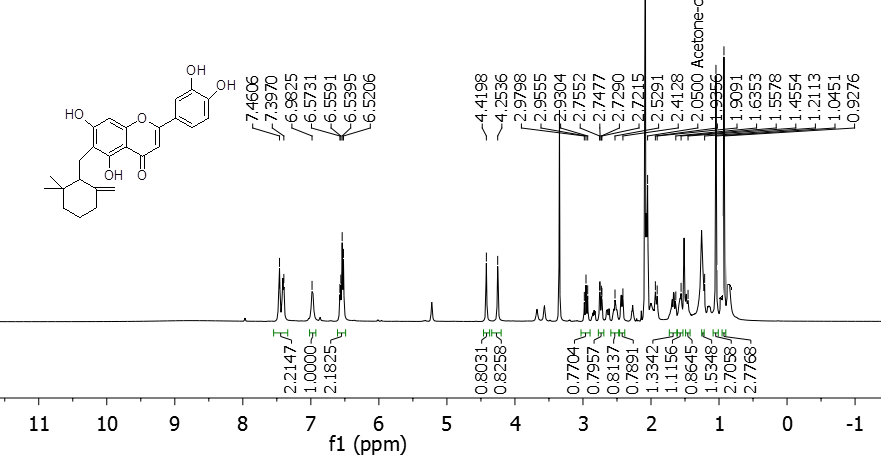


**Figure S1.** ^1^H NMR spectrum of compound **1** (500 MHz, Acetone-*d*_6_).


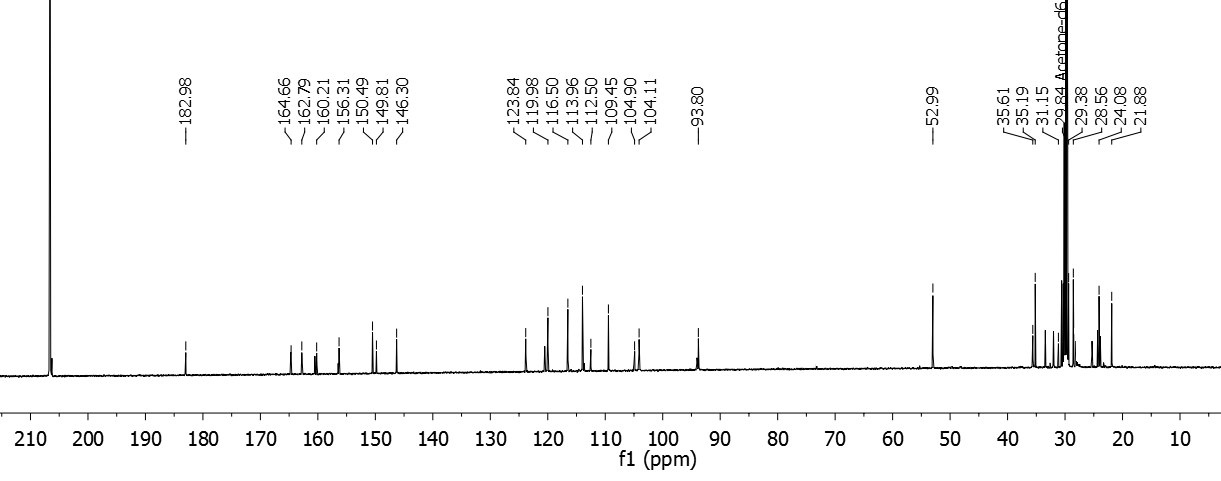


**F**i**gure S2.** ^13^C NMR spectrum of compound **1** (125 MHz, Acetone).


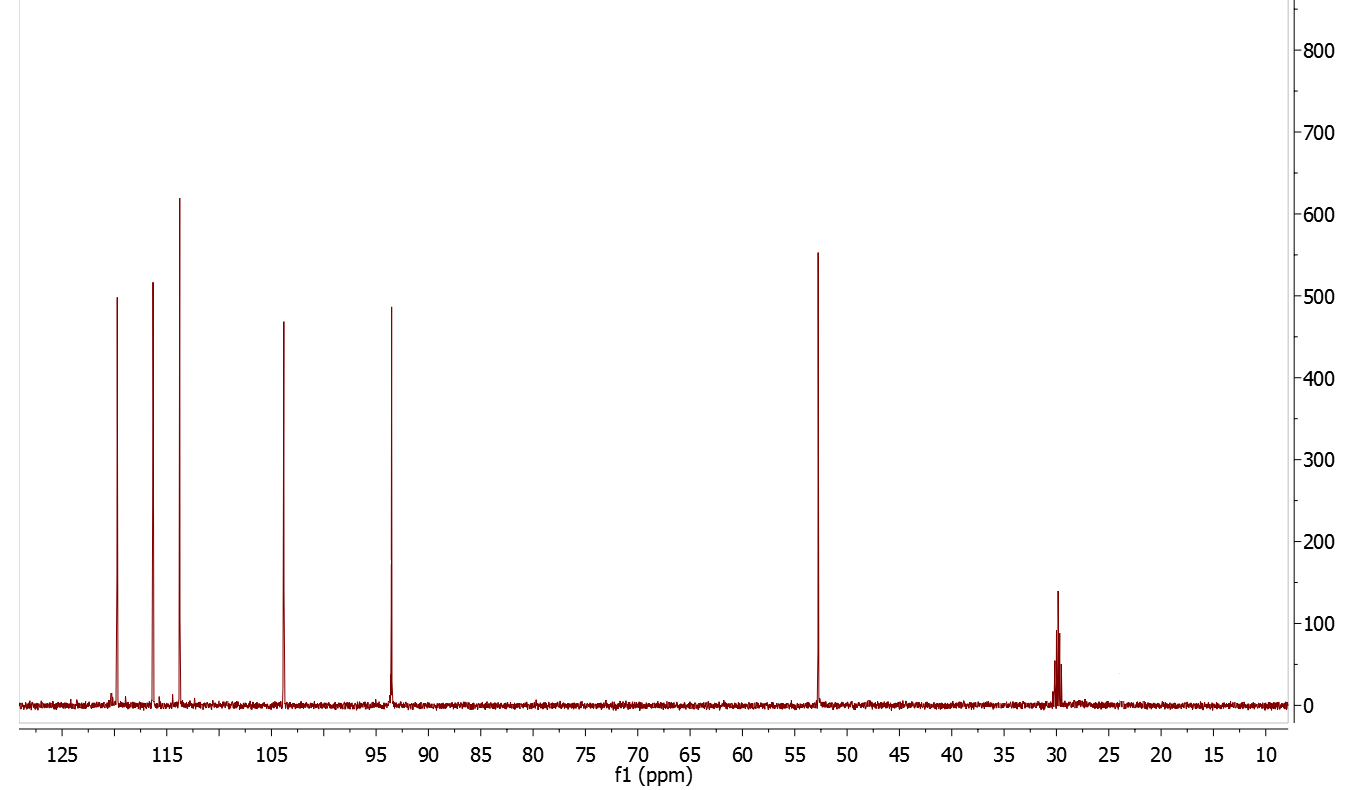


**Figure S3.** DEPT-90 spectrum of compound **1** (Acetone-*d*_6_).


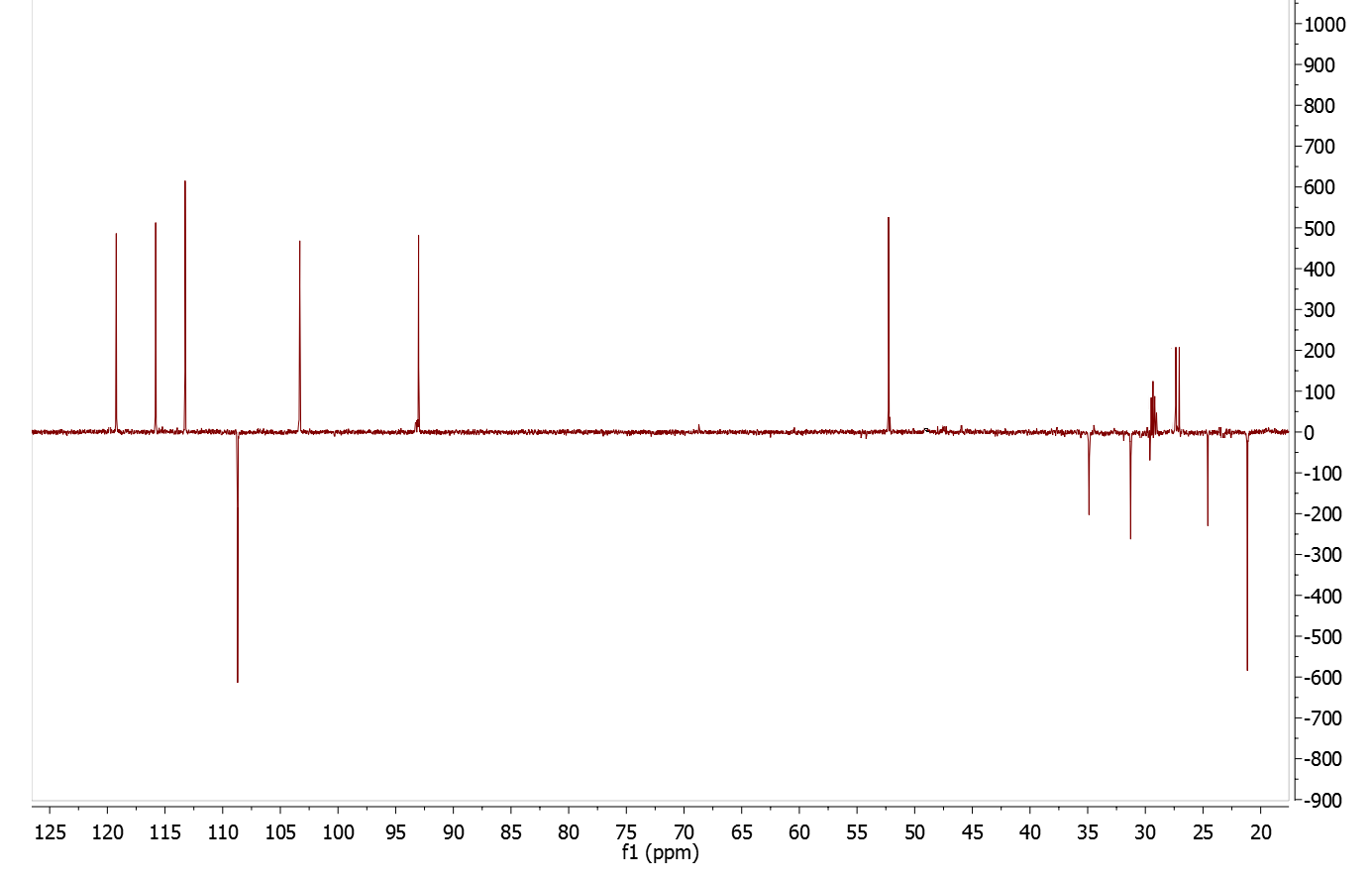


**Figure S4.** DEPT1-35 spectrum of compound **1** (Acetone-*d*_6_).


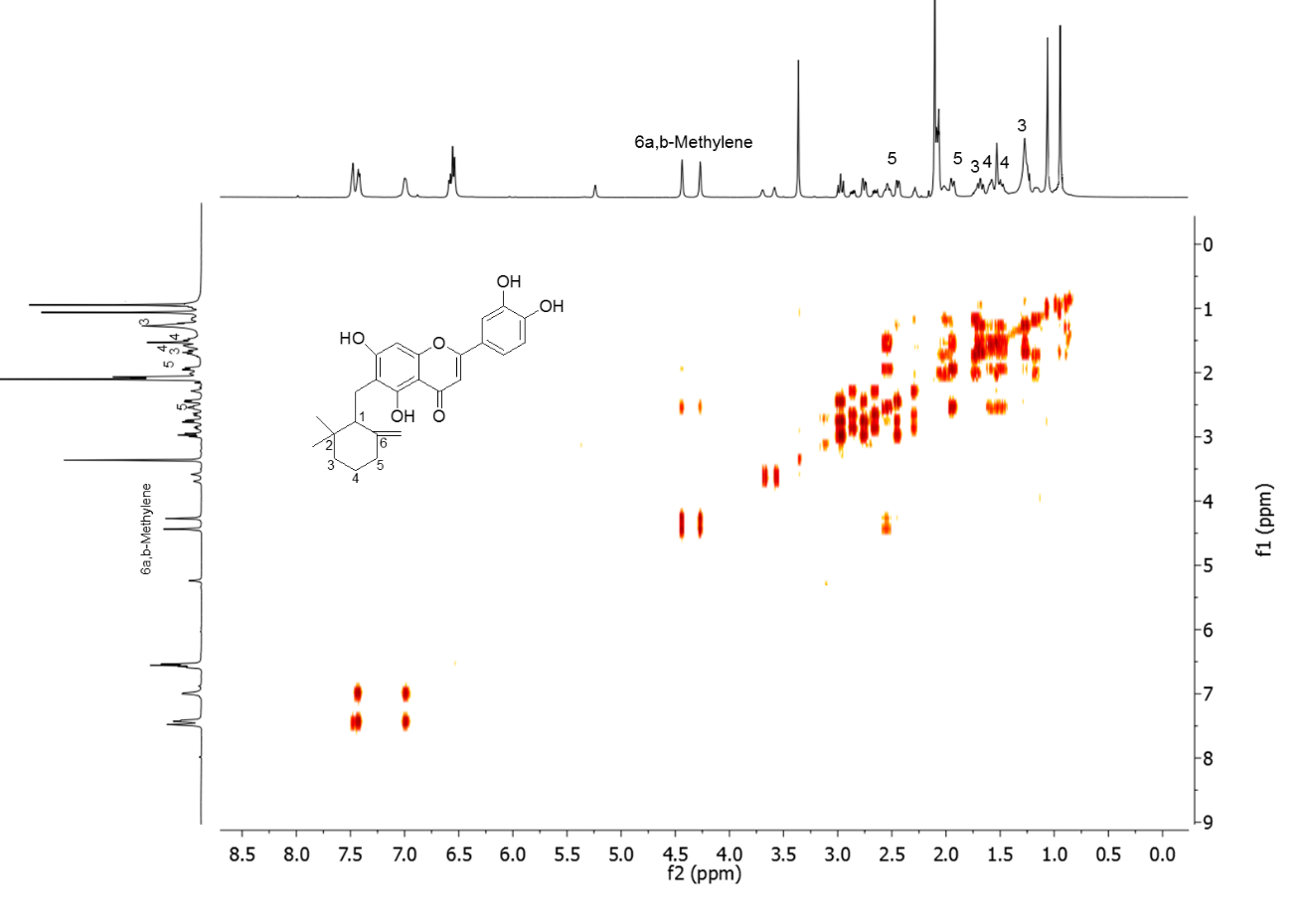


**Figure S5.** COSY spectrum of compound **1** (Acetone-*d*_6_).


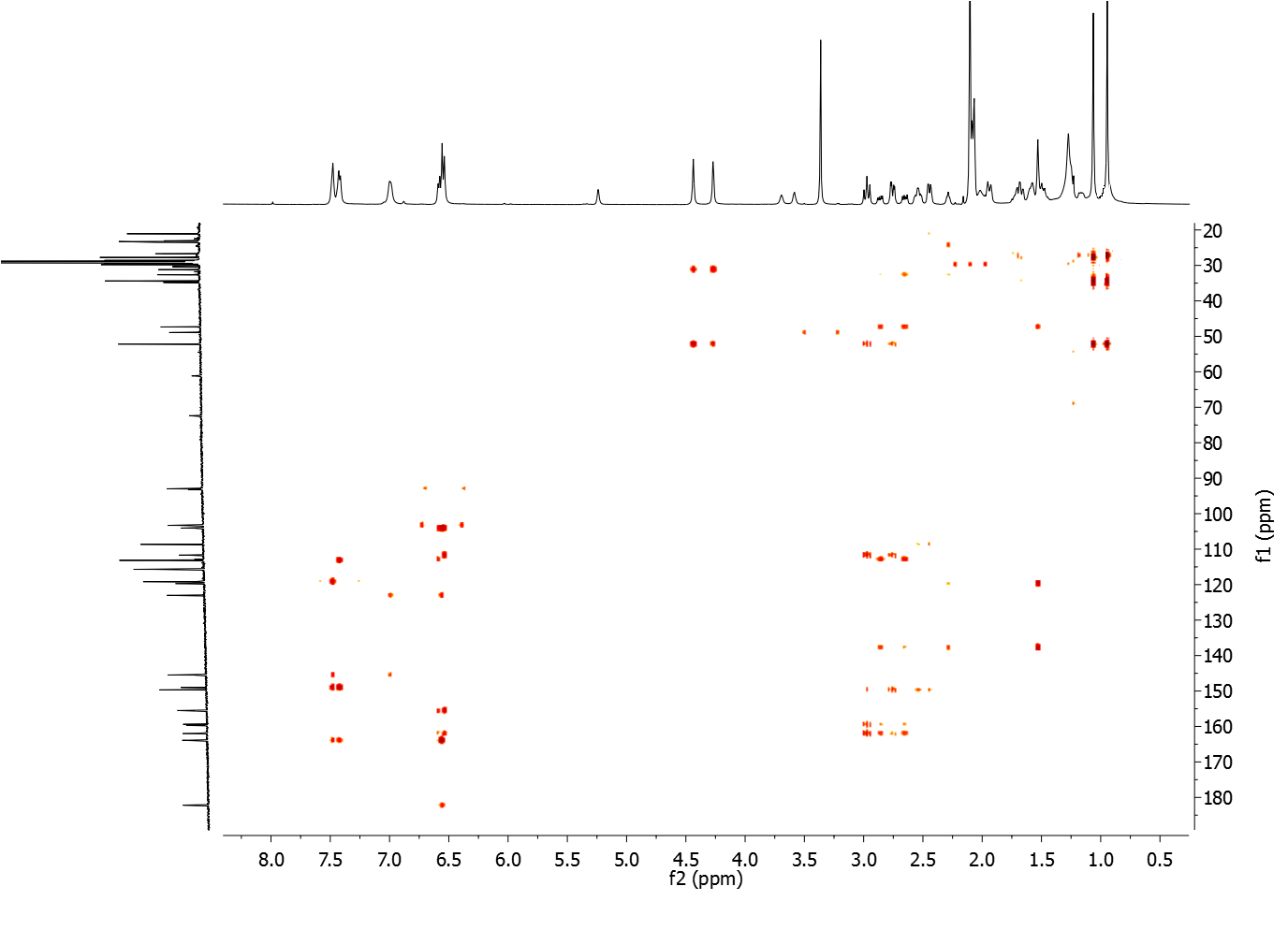
 **Figure S6.** HMBC spectrum of compound **1** (Acetone-*d*_6_).


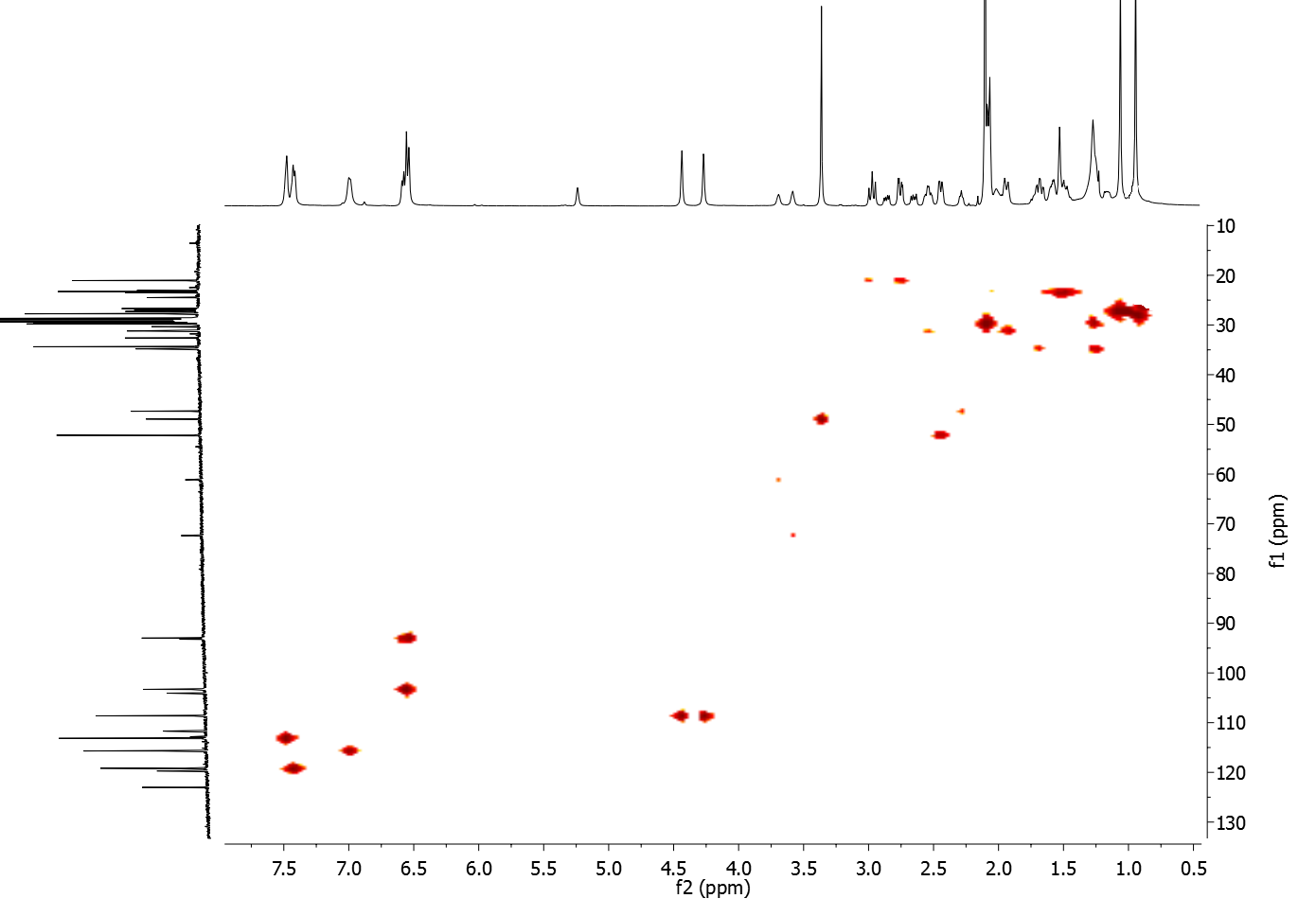


**Figure S7.** HMQC spectrum of compound **1** (Acetone-*d*_6_).

**
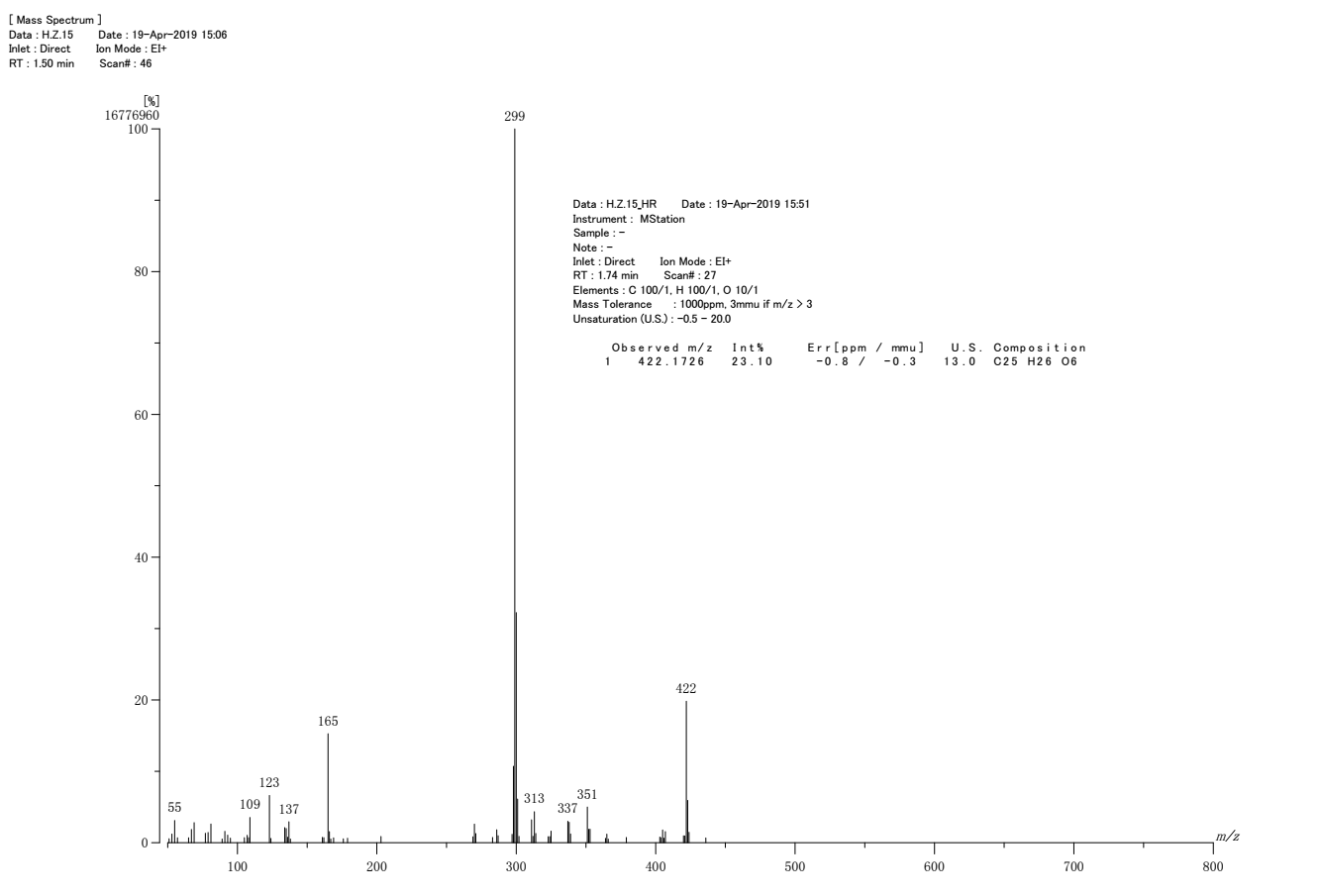
**

**Figure S8.** EIMS spectra and HREIMS data of compound **1.**


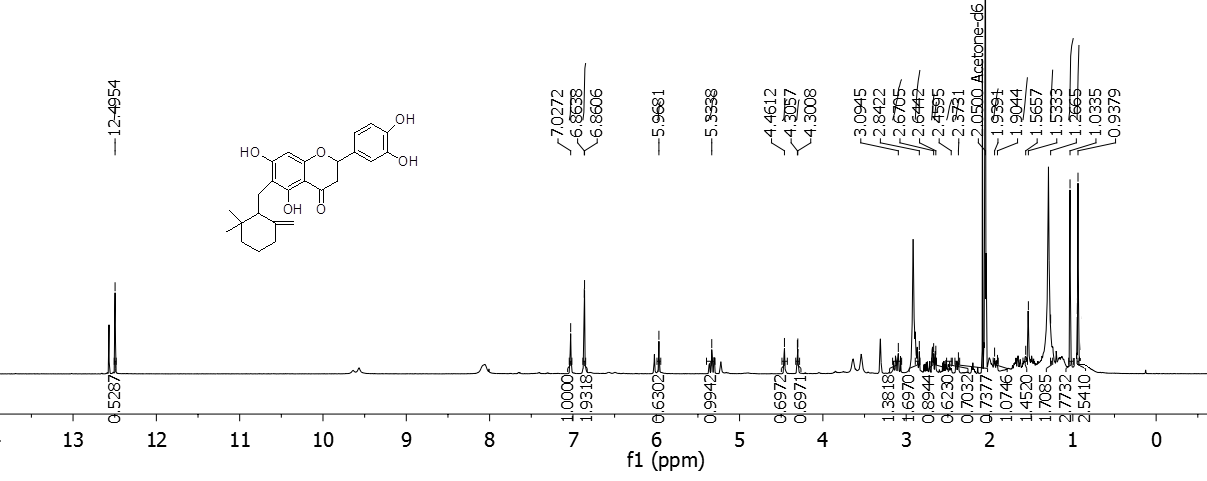


**Figure S9.** ^1^H NMR spectrum of compound **2** (500 MHz, Acetone-*d*_6_)


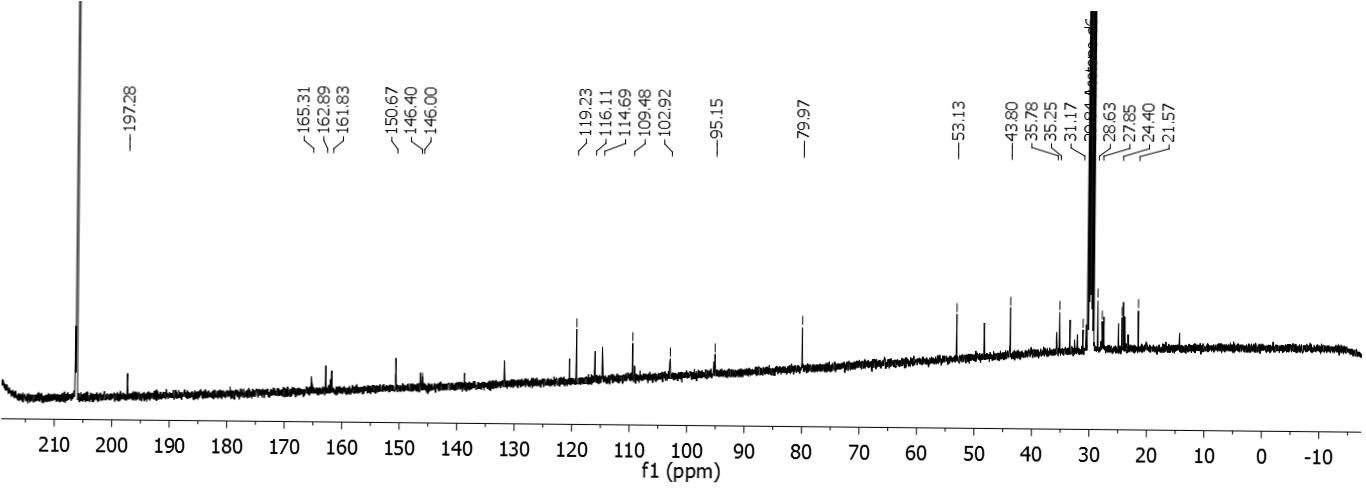


**Figure S10.** ^13^C NMR spectrum of compound **2** (125 MHz, Acetone-*d*_6_)


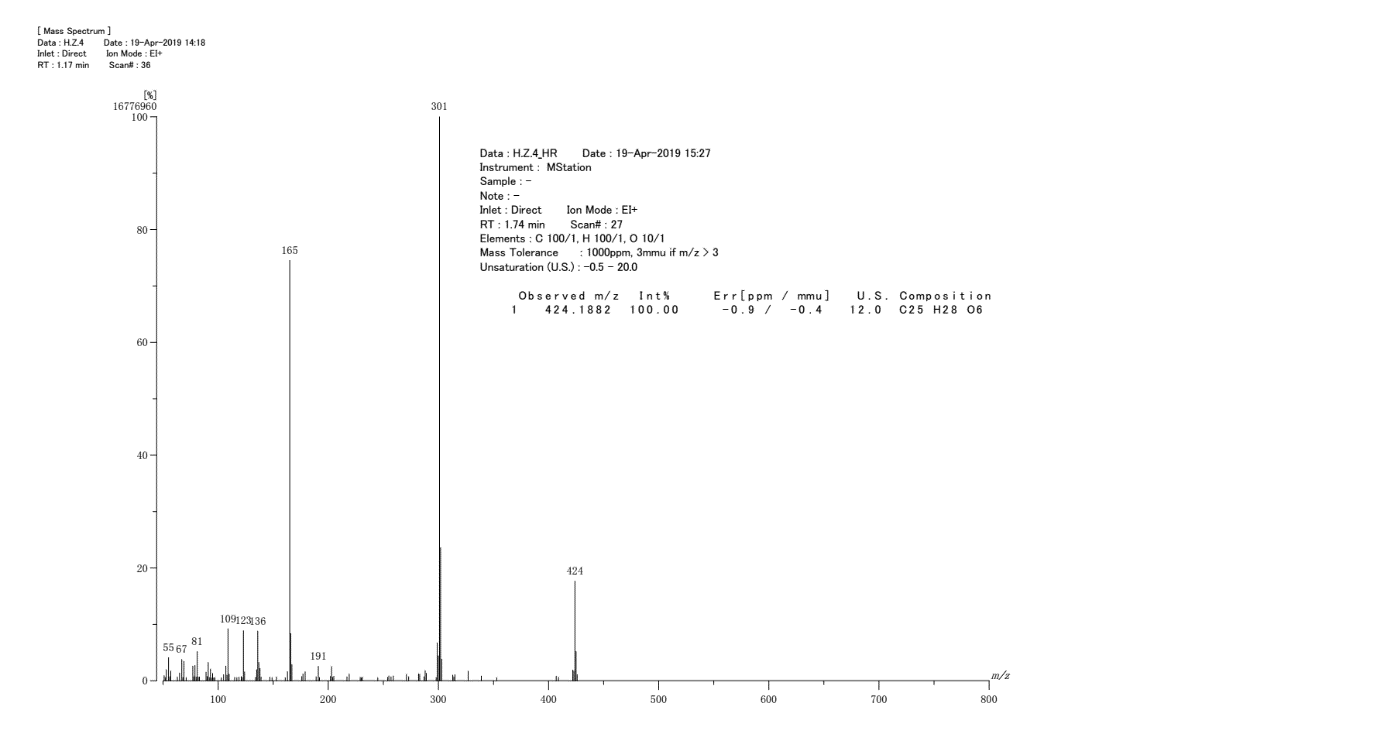


**Figure S11.** EIMS spectra and HREIMS data of compound **2.**

**
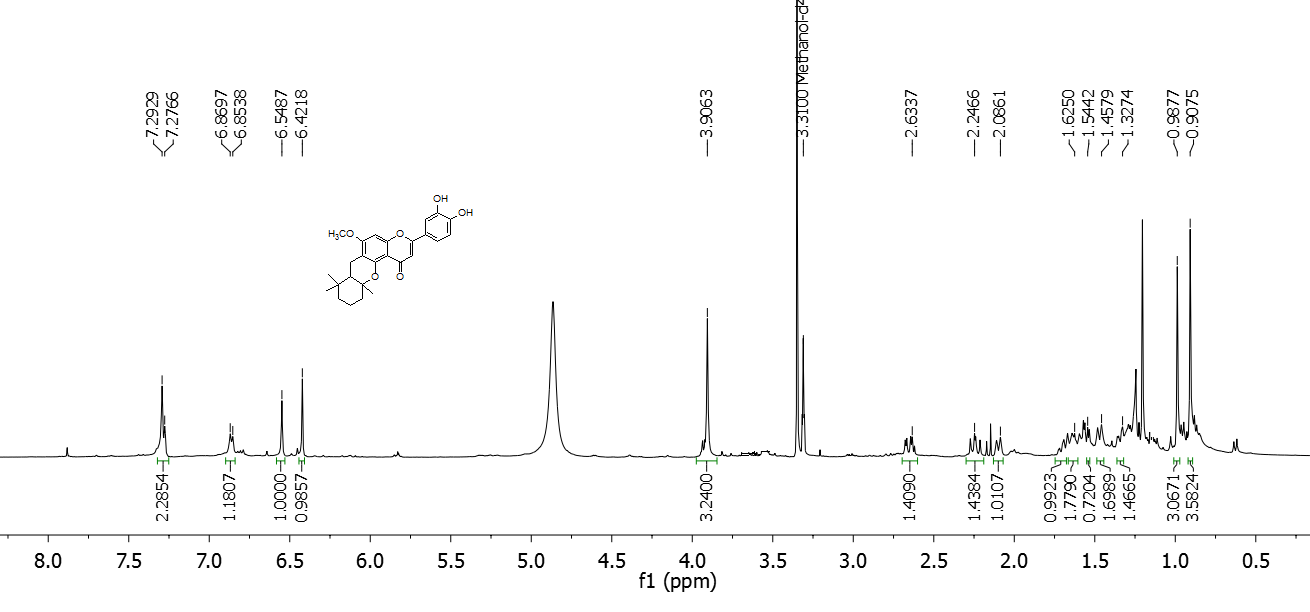
**

**Figure S15.** ^1^H NMR spectrum of compound **3** (500 MHz, MeOD)


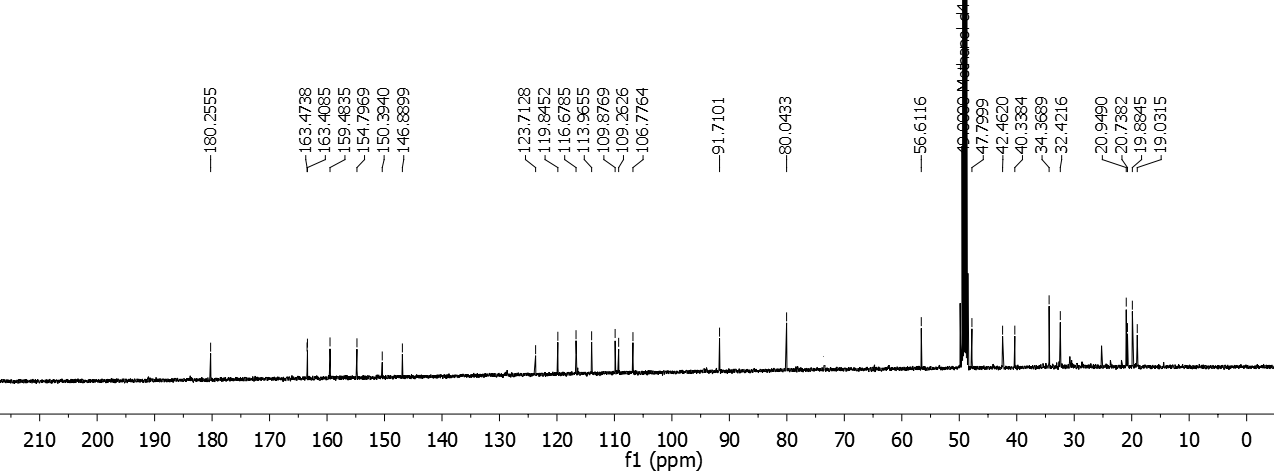


**Figure S16.** ^13^C NMR spectrum of compound **3** (125 MHz, MeOD).


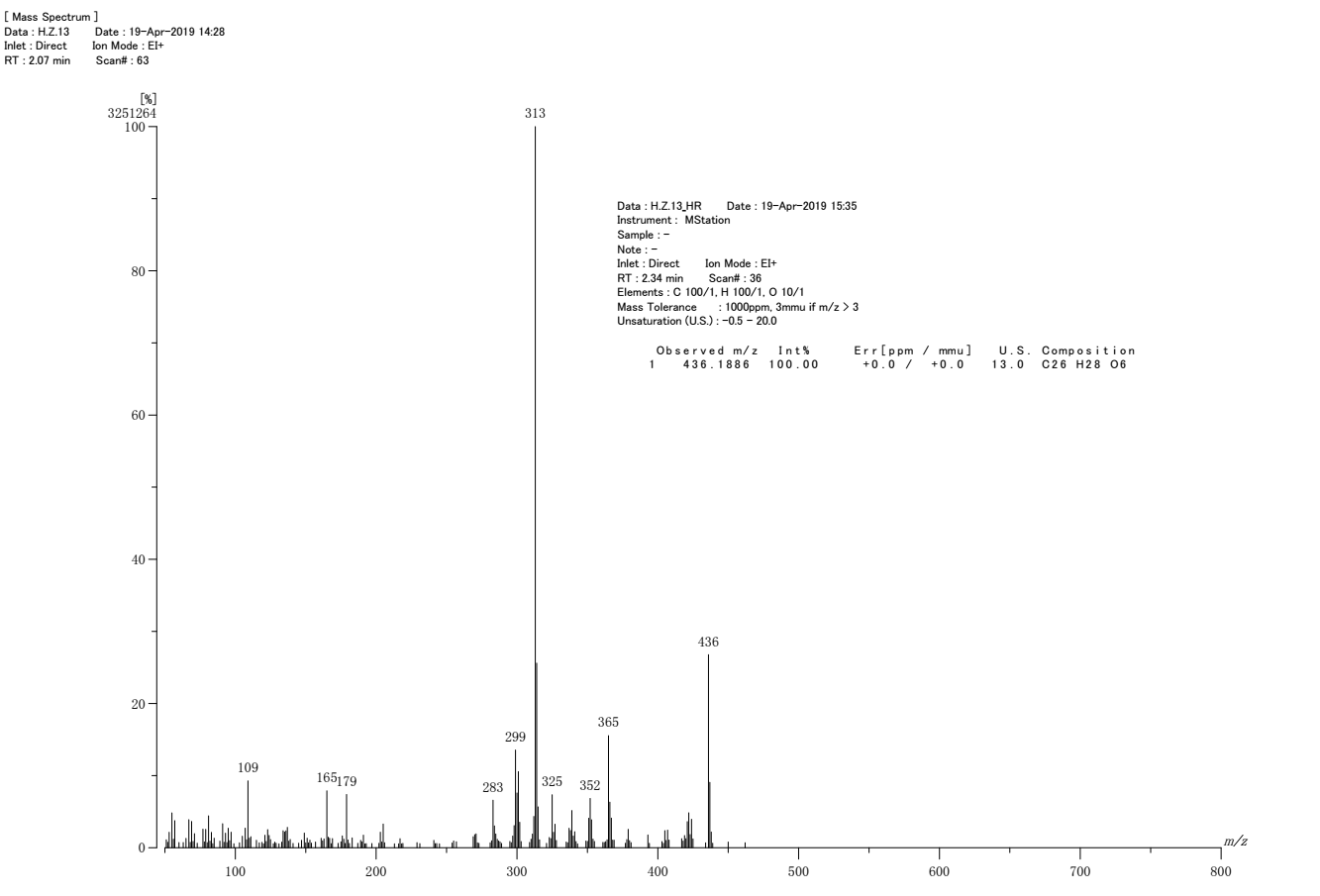


**Figure S17.** EIMS spectra and HREIMS data of compound **3.**

**
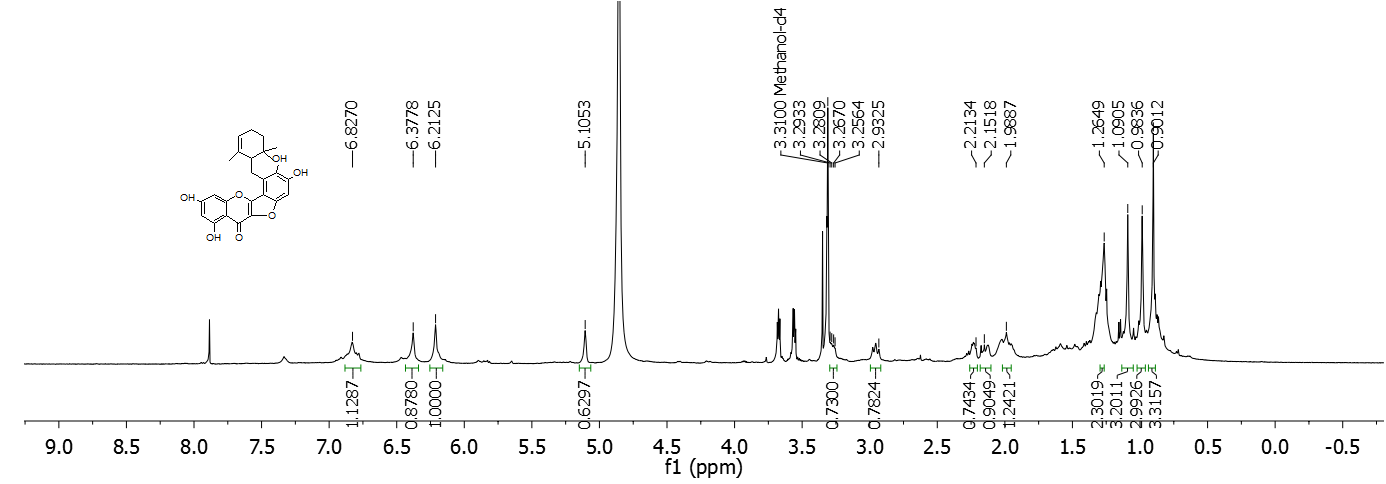
**

**Figure S21.** ^1^H NMR spectrum of compound **4** (500 MHz, MeOD)


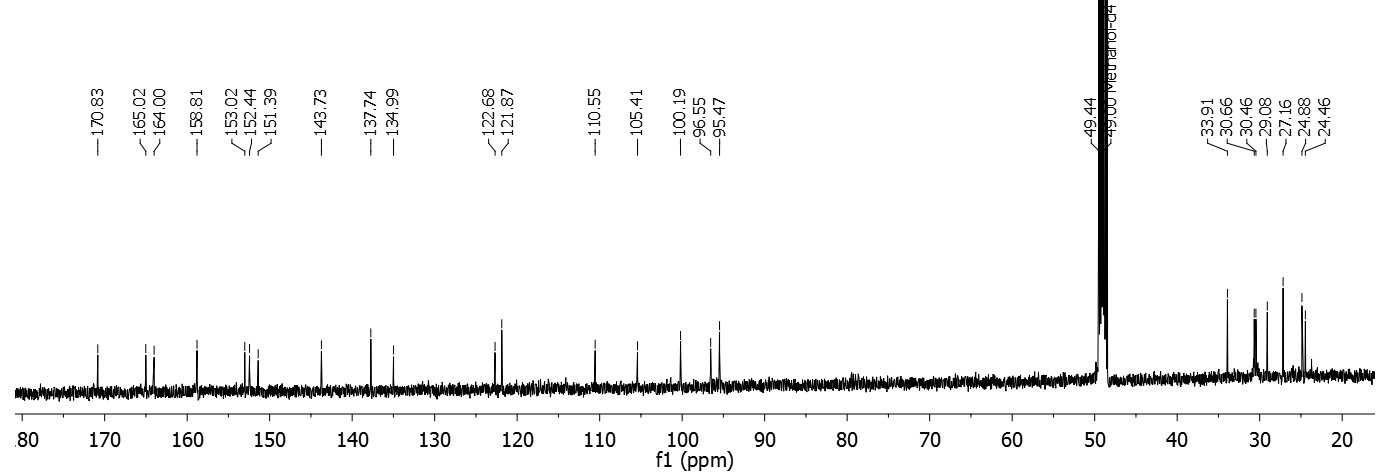


**Figure S12.** ^13^C NMR spectrum of compound **4** (125 MHz, MeOD).


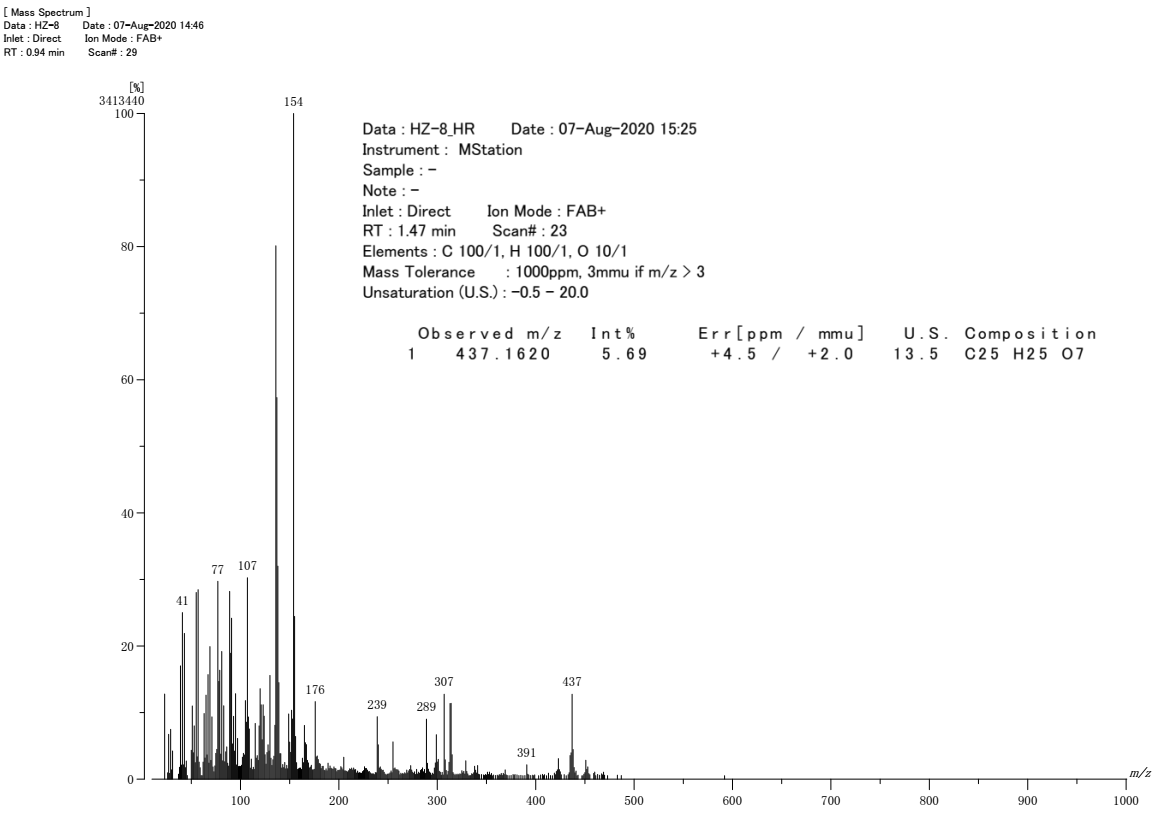


**Figure S23.** FABMS and HRFABMS spectra data of compound **4.**

**
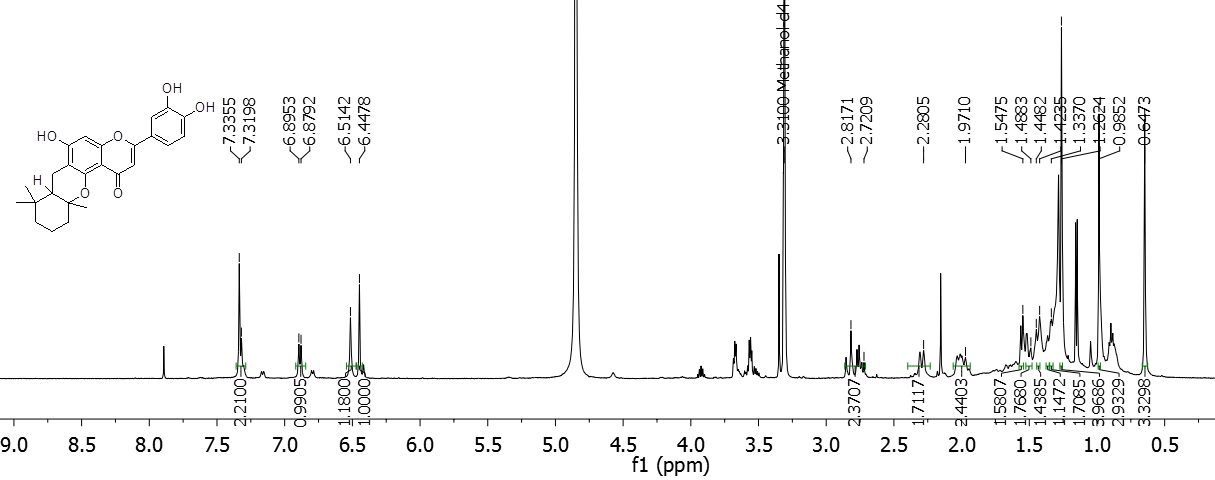
**

**Figure S12.** ^1^H NMR spectrum of compound **5** (500 MHz, MeOD)


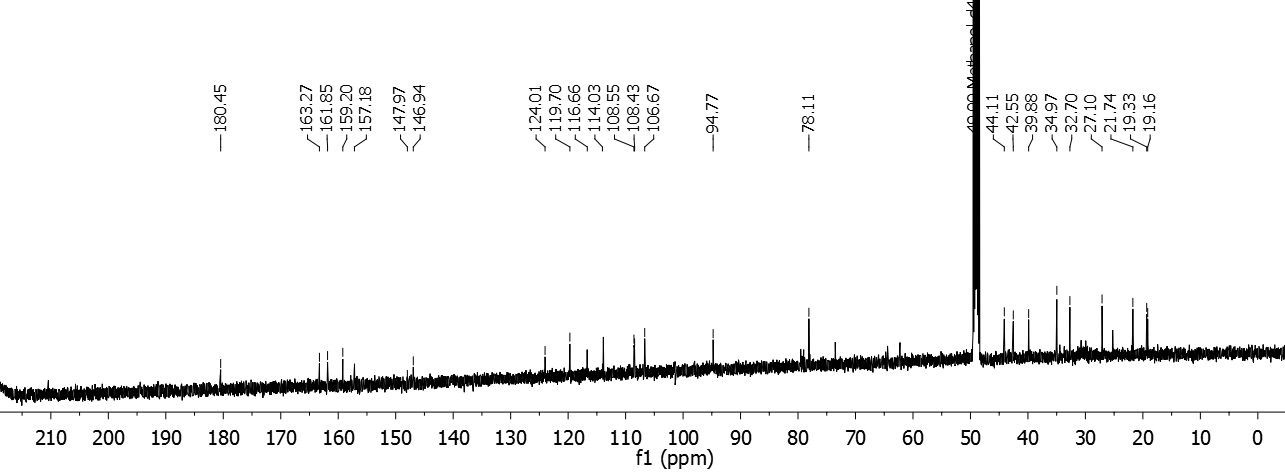


**Figure S13.** ^13^C NMR spectrum of compound **5** (125 MHz, MeOD).


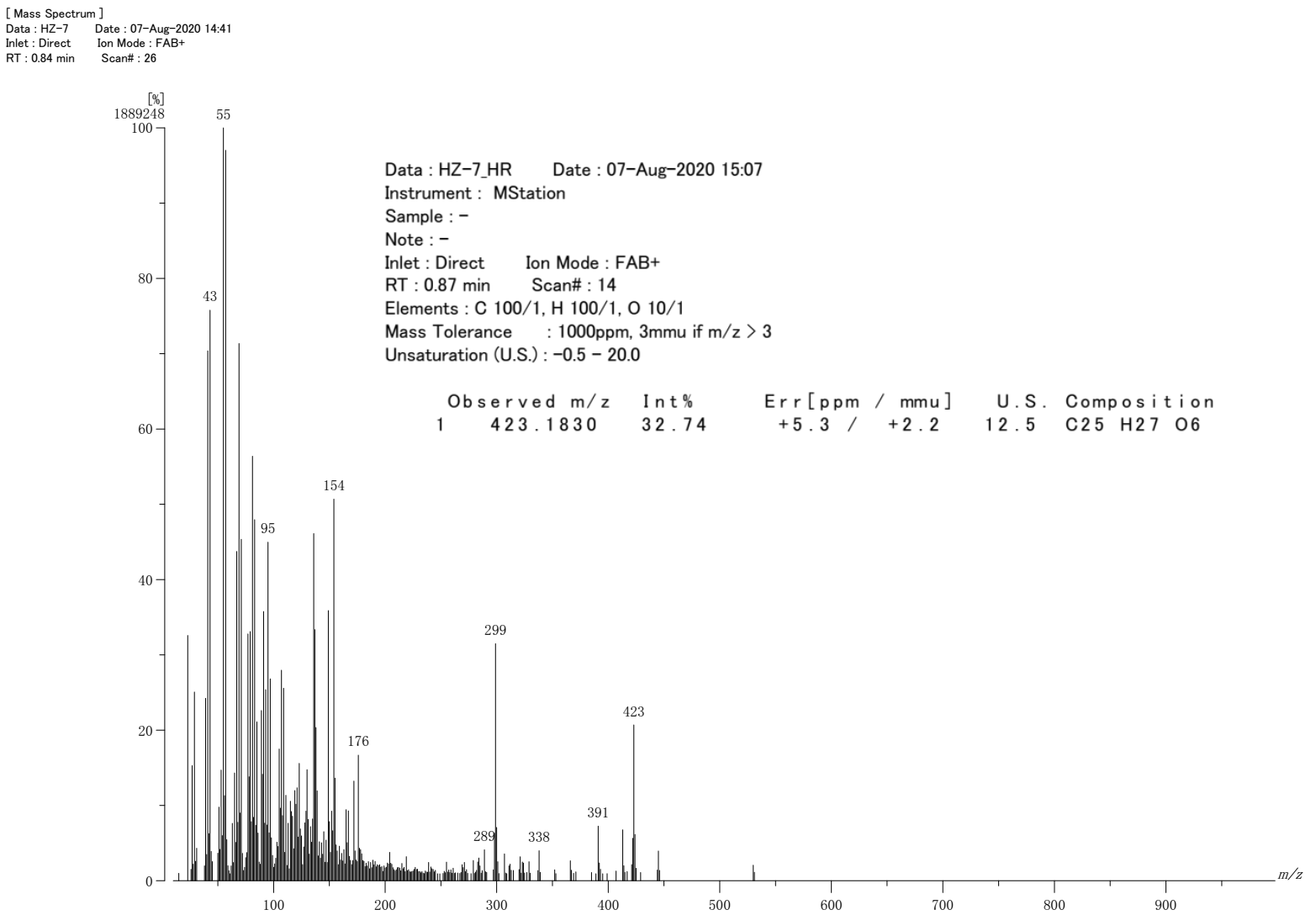


**Figure S14.** FABMS and HRFABMS spectra data of compound **5.**

**
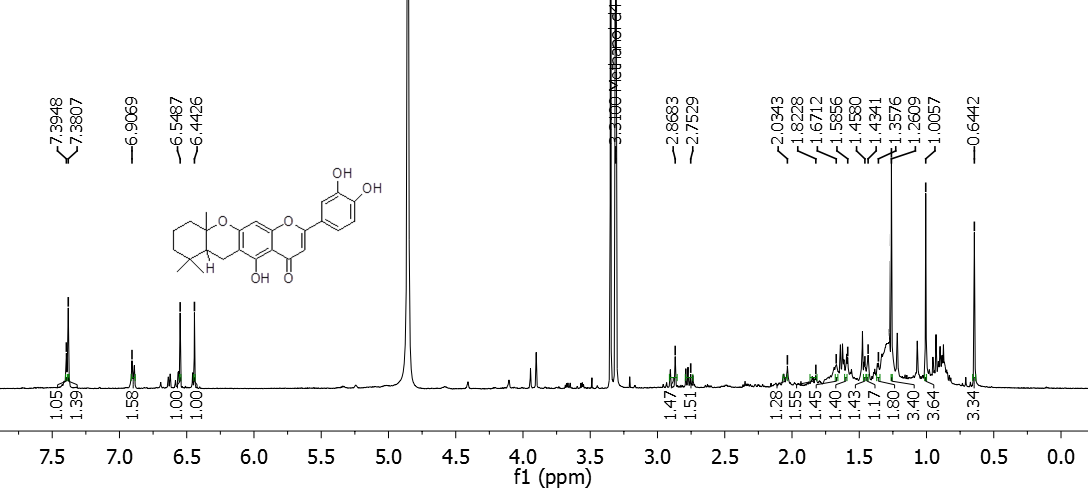
**

**Figure S18.** ^1^H NMR spectrum of compound **6** (500 MHz, MeOD).


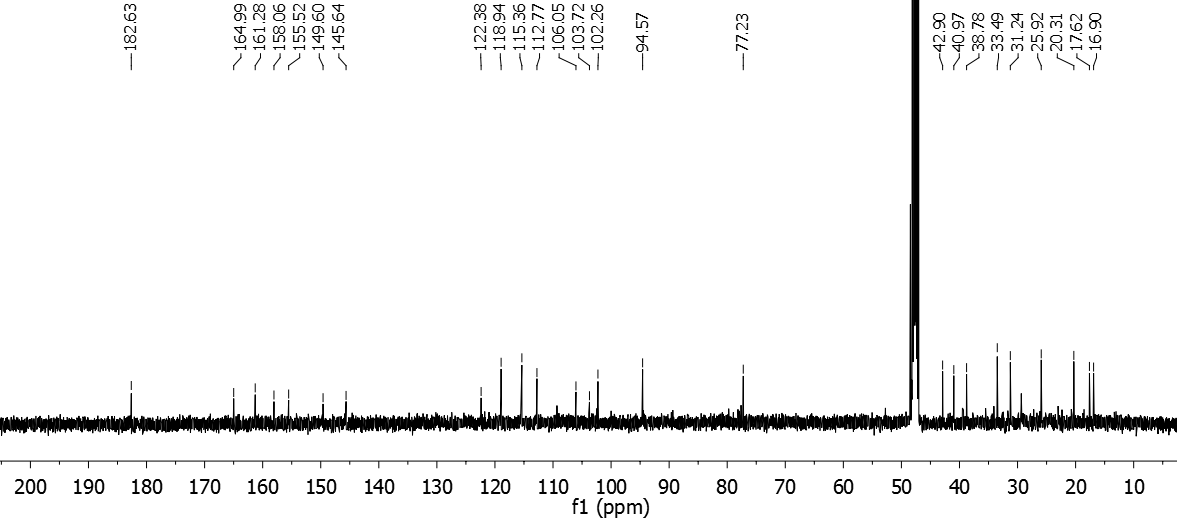


**Figure S19.** ^13^C NMR spectrum of compound **6** (125 MHz, MeOD).


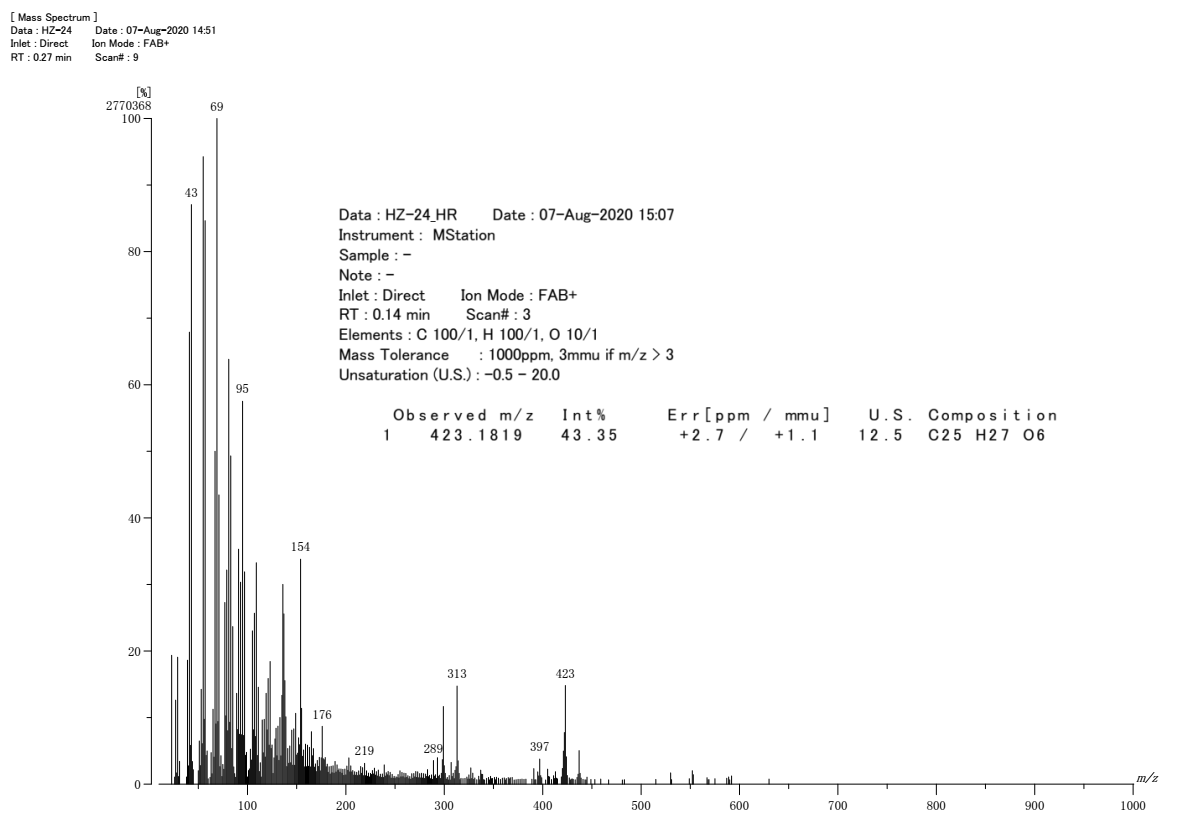


**Figure S20.** FABMS and HRFABMS spectra data of compound **6.**

**Figure S21.** Lineweaver burk and Dixon plots of compounds **2**, **3 and 4**

**Figure S22.** Lineweaver burk and Dixon plots of compounds **5 and 6**

**Figure S23.** The fluorescence spectra of compounds **3**, **4**, **5** & **6**, and the correlation between inhibitory potencies (IC_50_s) and Stern–Volmer constant (*K*_SV_)


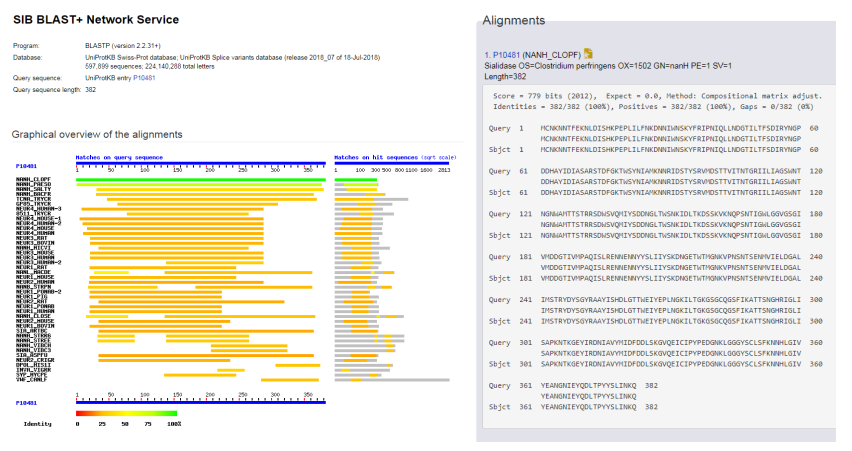


Figure S24. Fluorescence residues of neuraminidase from C. perfrigens. It was brought from ExPASy that is Swiss Institute of Bioinformatics. Trp31, 80, 118, 124, 135, 149, 172, 217, and 264. Tyr35, 57, 65, 82, 95, 141, 203, 204, 209, 246, 248, 251, 255, 267, 310, 318, 336, 347, 361, 369, 376, and 377. Phe8, 24, 36, 52, 76, 286, 322, and 352.

**Table S1.** UPLC-ESI-Q-TOF/MS characterization of compounds isolated from *H. zeylanica*.

| **№** | ***t*_R_ (min)** | **Formula** | **Neutral mass (Da)** | **Experimental *m*/*z* [M+H]^+^** | **Fragmental ion *m*/*z*** | **Identification** |
| --- | --- | --- | --- | --- | --- | --- |
| 1 | 9.12 | C_25_H_26_O_6_ | 422.1729 | 423.17932 | 299 | ugonin J |
| 2 | 11.98 | C_25_H_28_O_6_ | 424.1886 | 425.19443 | 301 | 2-(3,4-dihydroxyphenyl)-6-((2,2-dimethyl-6-methylenecyclo-hexyl)methyl)-5,7-dihydroxy-chroman-4-one |
| 3 | 10.95 | C_26_H_28_O_6_ | 436.1886 | 437.19477 | 313 | ugonin L |
| 4 | 11.10 | C_25_H_24_O_7_ | 436.1522 | 437.16000 |  | ugonin M |
| 5 | 9.01 | C_25_H_26_O_6_ | 422.1729 | 423.17906 | 299 | ugonin S |
| 6 | 11.80 | C_25_H_26_O_6_ | 422.1729 | 423.17945 | 299 | ugonin U |
